# Real-world safety of aliskiren in primary hypertension: A cross-database study

**DOI:** 10.1371/journal.pone.0346326

**Published:** 2026-04-03

**Authors:** Meirong Shan, Qian Guo, Ruofei Li, Ni Li, Yanhua Fu, Huanyu Qi, Ge Zhang, Qian Wang, Xingli Xu, Jinchuan Lai

**Affiliations:** 1 Department of Geriatrics, The First Affiliated Hospital of Zhengzhou University, Zhengzhou, Henan, China; 2 Department of Rhinology, The First Affiliated Hospital of Zhengzhou University, Zhengzhou, Henan, China; 3 Institute of Cardiovascular Diseases & Department of Cardiology, Sichuan Provincial People’s Hospital, School of Medicine, University of Electronic Science and Technology of China, Chengdu, China; 4 Department of Obstetrics and Gynecology, First People’s Hospital of Foshan, Foshan, Guangdong, China; 5 Department of Hepatology, Qilu Hospital of Shandong University (Qingdao), Qingdao, Shandong, China; 6 Department of Gastrointestinal Surgery, The First Affiliated Hospital of Zhengzhou University, Zhengzhou, Henan, China; 7 Department of Cardiology, The First Affiliated Hospital of Zhengzhou University, Zhengzhou, Henan, China; 8 Key Laboratory of Cardiac Injury and Repair of Henan Province; Henan Province Clinical Research Center for Cardiovascular Diseases, Zhengzhou, Henan, China; Kurume University School of Medicine, JAPAN

## Abstract

Hypertension is one of the main causes of cardiovascular diseases worldwide, affecting over one billion people. Although aliskiren offers a valuable option for inhibiting the renin-angiotensin system, its safety profile in the real world remains insufficiently explored, especially for rare or under-recognized adverse events (AEs), which have not been fully clarified. Therefore, leveraging large-scale post-marketing surveillance data is crucial for identifying rare AEs and guiding safer clinical practice. This study aims to elucidate pharmacovigilance signals associated with aliskiren (an antihypertensive drug) by systematically analyzing the characteristics of adverse events (AEs) from the U.S. Food and Drug Administration (FDA) Adverse Event Reporting System (FAERS) database and WHO-VigiAccess database, which provides a reliable scientific basis for clinical practice and regulatory decision-making. We conducted a retrospective quantitative analysis of aliskiren-related AE reports from the aforementioned two databases, employing the Proportional Reporting Ratio (PRR), Reporting Odds Ratio (ROR), Bayesian Confidence Propagation Neural Network (BCPNN), and Multi-item Gamma Poisson Shrinker (MGPS) algorithms for signal detection. The results indicate that there were 5,596 and 5,549 aliskiren-related reports in the FAERS and WHO-VigiAccess databases, respectively. The median duration of these AEs during the observation period was 62 days, with an interquartile range (IQR) of 7–282 days. In both databases, signals for aliskiren were distributed across 28 System Organ Classes (SOCs), among which investigations, cardiac disorders, renal and urinary disorders, vascular disorders, and metabolism and nutrition disorders exhibited significant signals based on specific criteria applied across the four algorithms. A total of 607 preferred terms (PTs) with significant disproportionality signals were detected using the four algorithms, including potential AEs not previously well-documented, such as palpitations, myalgia, proteinuria, muscular weakness, pulmonary edema, and pollakiuria. This study not only confirms the known adverse reactions of aliskiren but also uncovers new potential risks, highlighting the importance of strengthening drug safety monitoring to enhance therapeutic efficacy and reduce the risk of adverse reactions. It provides valuable safety insights for physicians considering the use of aliskiren in the management of primary hypertension.

## Introduction

Under the correct measurement method, hypertension can be diagnosed when the systolic blood pressure (SBP) value is ≥ 140 mmHg and/or the diastolic blood pressure (DBP) value is ≥ 90 mmHg [1]. Risk classification is conducted based on blood pressure levels, risk factors, target organ damage, and clinical complications, with higher risk grades indicating a greater likelihood of cardiovascular and cerebrovascular events (such as myocardial infarction and stroke) [2,3]. Hypertension is a major contributor to the development of stroke, myocardial infarction, heart failure, and chronic kidney disease [4]. As a result, it stands as the leading global cause of cardiovascular disease and premature death [4]. In recent years, hypertension has become increasingly common among younger populations. According to the World Health Organization (WHO), approximately 1/3 of adults worldwide suffer from hypertension, and this disease is responsible for about half of all stroke and heart disease deaths [5]. In 2019, it was estimated that 1.2 billion people worldwide suffered from hypertension, posing a significant challenge to the economic burden of global healthcare systems [6]. Therefore, strictly controlling blood pressure (BP) is of utmost importance, as it can significantly reduce the incidence of cardiovascular events and the overall mortality rate. Some patients can control their BP to a normal level by changing their lifestyle such as exercise and diet. However, the majority of patients cannot manage their BP well through non-pharmacological means.

**Fig 1:**
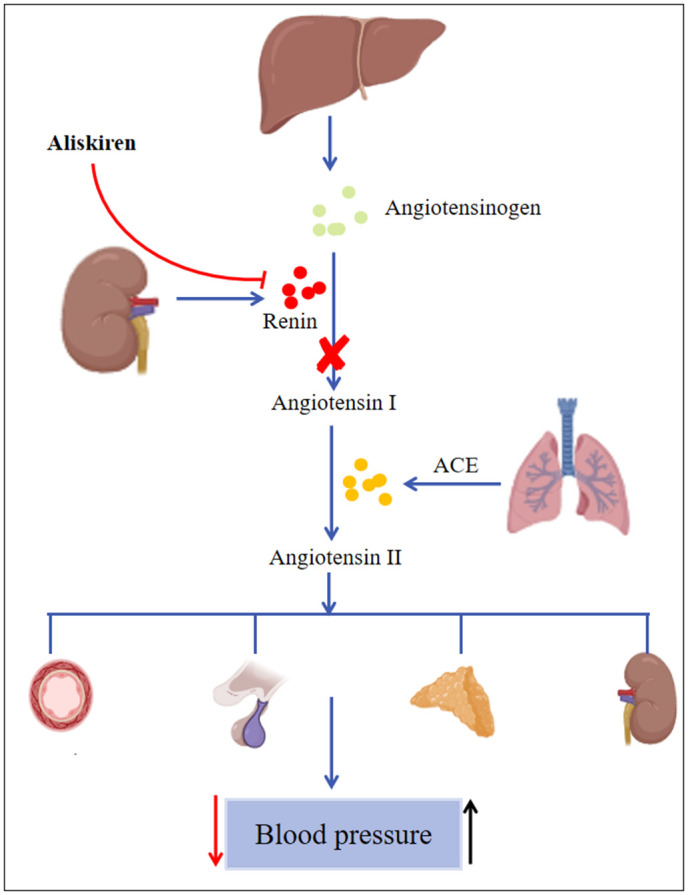
Pathway of the renin-angiotensin system (RAS) and the intervention mechanism of aliskiren. In the RAS pathway, aliskiren directly inhibits renin, blocking the conversion of angiotensinogen to angiotensin I (marked with a cross symbol), which leads to reduced angiotensin II production and ultimately lowers blood pressure. ACE, angiotensin-converting enzyme.

The current drugs used for treating hypertension mainly include five categories: beta-blockers, angiotensin-converting enzyme inhibitors (ACEI), angiotensin II receptor antagonists (ARB), calcium channel blockers, and diuretics [7]. Aliskiren is the first oral renin inhibitor approved for clinical treatment [8]. It is mainly metabolized and excreted via the feces in its original form, with only a very small portion being excreted through the kidneys. It directly inhibits the activity of renin, thereby blocking the activation of the entire renin-angiotensin-aldosterone system (RAAS) [8]. This further reduces the production of angiotensin II and aldosterone, leading to vasodilation, decreased sympathetic nerve activity, and increased sodium excretion, thereby lowering BP (Fig 1) [9]. Aliskiren has been proven to be effective in lowering BP whether used alone or in combination [10]. It is particularly valuable for patients who do not respond to or cannot tolerate other antihypertensive agents [10]. In the ALiskiren Observation of Heart Failure Treatment (ALOFT) study, it was found that regardless of whether patients received ACEIs (or ARBs) treatment or not, aliskiren was able to exert neurohumoral inhibitory effects on patients with heart failure (HF) [11,12]. And the AVOID sub-study demonstrated that adding aliskiren to losartan-based antihypertensive therapy significantly reduced urinary aldosterone excretion and slowed the decline of renal function [13].

FAERS database (the world’s largest open drug monitoring database) [14] and the World Health Organization’s VigiAccess database can conduct population-level aggregation, analysis, and evaluation of adverse drug reactions (ADRs) and drug-related safety issues. The FAERS database is regularly updated and publicly accessible, which helps to identify new adverse reaction signals. This article utilizes advanced data mining of AE reports from the FAERS and VigiAccess databases to establish an evidence base for personalized treatment decisions. It provides clinicians, patients, and regulatory agencies with deeper insights into the safety characteristics of the drugs. By integrating real-world evidence, we have depicted the risk-benefit balance in aliskiren treatment for the hypertensive patient population, providing operational guidance for optimizing clinical safety protocols.

## Method

### Data sources

This study utilized FAERS data, covering reports from 2004Q1 to 2025Q3, as well as the VigiAccess database, which includes all historical records up to September 30, 2025. FAERS data extraction was performed using OpenVigil 2.1 (a publicly accessible online tool for disproportionation analysis), and aliskiren was retrieved as the main suspected drug. AEs were classified according to the Medical Dictionary for Regulatory Activities (MedDRA) hierarchy, including preferred terms (PTs) and system organ classes (SOCs). In addition, the VigiAccess database, which is the World Health Organization’s (WHO) global database of individual case safety reports (ICSRs), was also queried to supplement the international safety data related to aliskiren. And duplicate reports were removed following the FDA’s recommended deduplication method, retaining the most recent FDA_DT for the same CASEID in the FAERS database. Reports where aliskiren was listed only as a concomitant or interacting drug were excluded from the primary signal detection analysis to minimize confounding. Cases with missing critical information (e.g., age or gender) were excluded from stratified demographic analyses but were retained for overall signal detection. For the VigiAccess database, we included all available reports up to September 30, 2025, and applied the same drug role and data completeness criteria where applicable.

**Fig 2:**
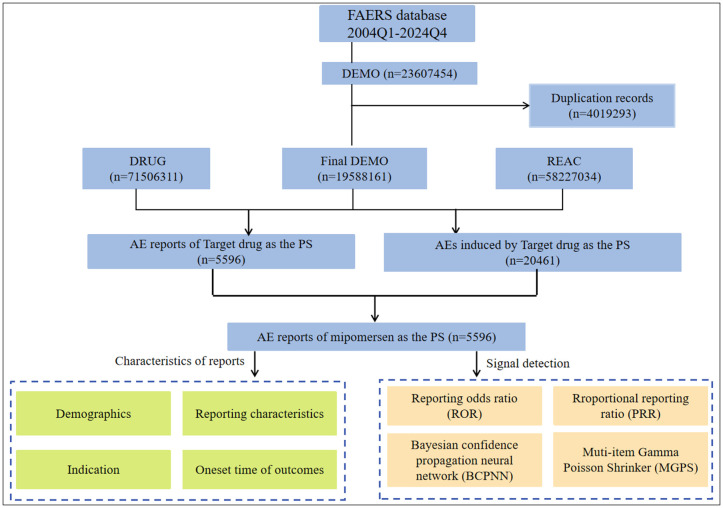
Flowchart illustrating the adverse events analysis process for aliskiren using the FAERS database.

In this study, the FAERS database contained a total of 19,588,161 AE reports, and 5,596 of these were attributed to aliskiren. From the FAERS data, we extracted 582,270,340 records, among which 20461 cases were related to the use of aliskiren by the general population (as shown in Fig 2).

### Data standardization

The original data obtained from the FAERS and VigiAccess databases were processed for record deduplication and standardization of AE terms. All reported AEs were standardized using MedDRA (version 26.0). Each AE was assigned a unique PT code and classified into different SOCs according to the hierarchical classification system to reflect the affected organ systems. For ambiguous terms, two independent reviewers made manual determinations to ensure compliance with MedDRA standards.

### Statistical analysis

We conducted a retrospective quantitative analysis of AE reports related to aliskiren extracted from the FAERS and VigiAccess databases. We conducted a disproportionality analysis using ROR [15], PRR [16], MGPS [17], and BCPNN [18] to detect and visualize potential drug-event associations. S1 Table provides a detailed description of the two-by-two-column union table. The signals were defined as ROR > 1 (lower limit of 95% CI > 1), PRR ≥ 2 (chi-square value≥4), IC025 ＞ 0， and EBGM＞2 (S2 Table).

## Results

### Descriptive analysis of AE report characteristics

**Fig 3:**
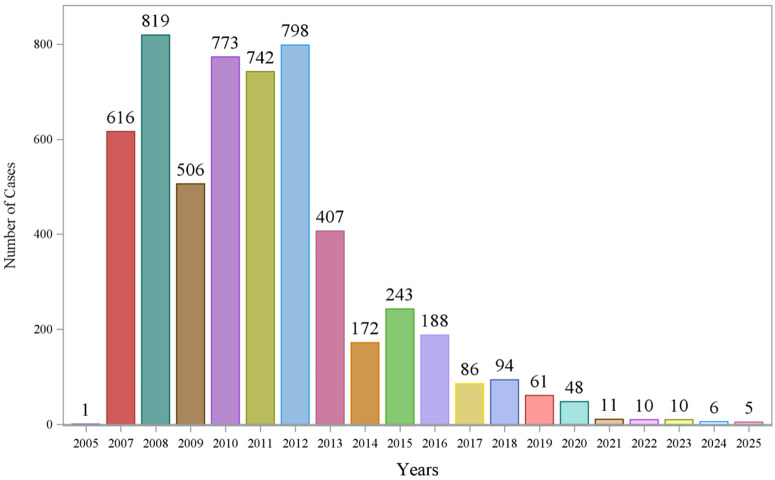
Annual distribution and trends of aliskiren adverse event reports.

The descriptive variable results in the FAERS and VigiAccess databases are shown in Tables 1 and 2. The FAERS and VigiAccess databases respectively contain 5,596 and 5,549 AE reports related to aliskiren. In terms of gender distribution, the proportion of females in both databases is higher than that of males. Regarding age distribution, nearly half of the patients in both databases lack age information. Among the existing data, patients aged 65 and above are more likely to report AEs, followed by those in the 45–64 age group, while patients aged 44 and below have the lowest reporting rate. In the FAERS database, the first reported year of aliskiren was 2005, and in the VigiAccess database, it was 2006. The Americas reported the most cases. The AE reports of aliskiren showed a decreasing trend year by year as shown in Fig 3. In the FAERS database, the most reported population was physicians, and the distribution of severe outcome percentages was shown as follows: 81.47% for Non-Serious and 18.53% for Serious. Among the severe outcomes, hospitalization had the highest proportion (31%). In both databases, Investigations were the most reported adverse reaction category.

**Table 1:** Clinical characteristics and essential demographic of AE reports related to aliskiren from the FAERS database (Q4 2023–Q4 2024).

Characteristics	FAERS database	
	Number of cases	Case proportion (%)
Number of events	5596	
Sex		
Female	2701	48.27
Male	2430	43.42
Missing	465	8.31
Age		
<18	6	0.11
18-44	139	2.48
45-64	944	16.87
≥65	1747	31.22
Missing	2760	49.32
Top 6 reporting countries		
America	2085	37.26
Germany	715	12.78
Japan	414	7.4
Canada	346	6.18
France	322	5.75
Brazil	250	4.47
Reporter		
Consumer	1713	30.61
Pharmacist	247	4.41
Physician	2919	52.16
Lawyer	24	0.43
Other health-professional	517	9.24
Missing	176	3,15
AE severity		
Non-Serious	4559	81.47
Serious	1037	18.53
Serious outcome		
Life-Threatening	277	4.95
Hospitalization	1735	31.00
Disability	98	1.75
Death	548	9.79
Congenital Anomaly	8	0.14
Required Intervention to Prevent Permanent Impairment/Damage	4	0.07
Other Serious Medical Events	3080	55.04

**Table 2:** Clinical Characteristics of adverse events for aliskiren in the VigiAccess database.

Characteristics	VigiAccess database	
	Number of cases	Case proportion (%)
Number of events	5549	
Sex		
Female	2792	50.32
Male	2175	39.2
Unknown	582	10.49
Age		
<18	17	0.31
18-44	176	3.17
45-64	978	17.62
≥65	1816	32.73
Unknown	2562	46.17
Continent		
Americas	3195	57.58
Asia	269	4.85
Europe	2082	37.52
Africa	3	0.05
Report year		
2006	3	0.05
2008	973	17.53
2009	752	13.55
2010	883	15.91
2011	688	12.4
2012	886	15.97
2013	294	5.3
2014	369	6.65
2015	278	5.01
2016	129	2.32
2017	59	1.06
2018	69	1.24
2019	67	1.21
2020	25	0.45
2021	28	0.5
2022	25	0.45
2023	8	0.14
2024	13	0.23

### Distribution of AEs at the SOCs level in two databases

**Fig 4:**
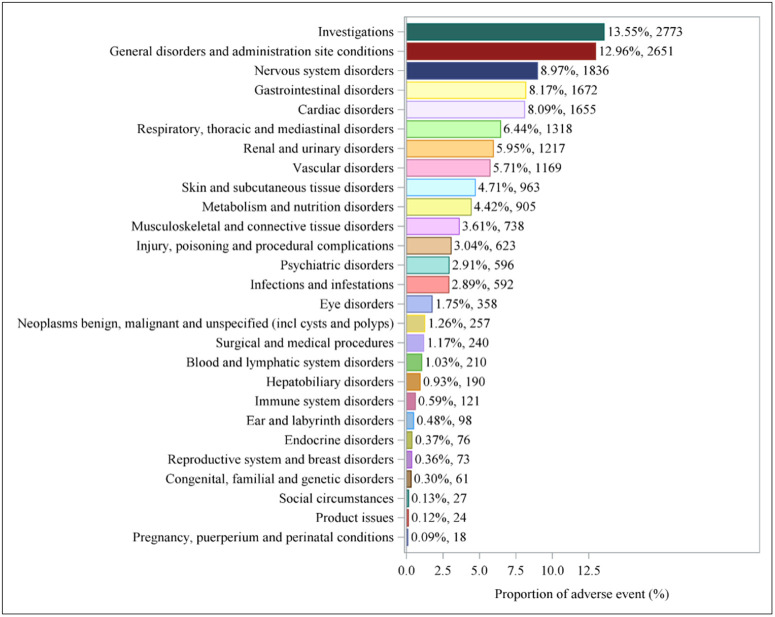
Proportion and frequency of adverse events by system organ class for aliskiren.

The AE signals of aliskiren were classified according to the SOC system. Fig 4 shows the percentage of positive signals of AEs related to aliskiren. Tables 3 and 4 respectively provide detailed information on the specific signal intensities of aliskiren in the FAERS and VigiAccess databases at the SOCs level. Among the 28 SOC systems in the FAERS database, the SOC systems that simultaneously meet the four specific criteria include investigations (n = 2773, ROR (95% CI): 2.42 (2.32, 2.52)), cardiac disorders (n = 1655, ROR (95% CI): 3.31 (3.15, 3.48)), renal and urinary disorders (n = 1217, ROR (95% CI): 3.30 (3.12, 3.50)), vascular disorders (n = 1169, ROR (95% CI): 2.80 (2.64, 2.97)), and metabolism and nutrition disorders (n = 905, ROR (95% CI): 2.09 (1.96, 2.24)). In the VigiAccess database, investigations (n = 2495, ROR (95% CI): 2.54 (2.43, 2.65)), cardiac disorders (n = 1102, ROR (95% CI): 2.84 (2.67, 3.02)), renal and urinary disorders (n = 864, ROR (95% CI): 3.27 (3.06, 3.50)), vascular disorders (n = 1129, ROR (95% CI): 3.25 (3.06, 3.45)), and metabolism and nutrition disorders (n = 701, ROR (95% CI): 2.35 (2.18, 2.53)) have statistically significant signals.

**Table 3:** Signal strength of aliskiren related adverse events across system organ classes (SOCs) in the FDA Adverse Event Reporting System database.

SOCs	Number of cases	ROR (95% CI)	PRR (95% CI)	chi-square	IC (IC025)	EBGM (EBGM05)
Investigations*	2773	2.42(2.32,2.52)	2.22(2.15,2.30)	1989.461072	1.15(1.09)	2.22(2.14)
General disorders and administration site conditions	2651	0.70(0.68,0.73)	0.74(0.72,0.77)	285.0873213	−0.43(−0.49)	0.74(0.71)
Nervous system disorders	1836	1.07(1.02,1.13)	1.07(1.02,1.11)	8.190709397	0.09(0.02)	1.07(1.02)
Gastrointestinal disorders	1672	0.96(0.91,1.01)	0.96(0.92,1.01)	2.877466698	−0.06(−0.13)	0.96(0.91)
Cardiac disorders*	1655	3.31(3.15,3.48)	3.12(2.98,3.27)	2451.562123	1.64(1.57)	3.12(2.97)
Respiratory, thoracic and mediastinal disorders	1318	1.40(1.32,1.48)	1.37(1.30,1.44)	138.8678576	0.46(0.37)	1.37(1.30)
Renal and urinary disorders*	1217	3.30(3.12,3.50)	3.16(3.00,3.34)	1834.194967	1.66(1.57)	3.16(2.98)
Vascular disorders*	1169	2.80(2.64,2.97)	2.70(2.55,2.85)	1274.017503	1.43(1.34)	2.70(2.54)
Skin and subcutaneous tissue disorders	963	0.86(0.81,0.92)	0.87(0.81,0.92)	21.20187106	−0.21(−0.30)	0.87(0.81)
Metabolism and nutrition disorders*	905	2.09(1.96,2.24)	2.04(1.92,2.18)	492.1940563	1.03(0.93)	2.04(1.91)
Musculoskeletal and connective tissue disorders	738	0.69(0.64,0.74)	0.70(0.65,0.75)	99.22105672	−0.51(−0.62)	0.70(0.65)
Injury, poisoning and procedural complications	623	0.27(0.25,0.29)	0.29(0.27,0.31)	1227.322594	−1.80(−1.91)	0.29(0.27)
Psychiatric disorders	596	0.51(0.47,0.55)	0.52(0.48,0.57)	273.2234389	−0.93(−1.05)	0.52(0.48)
Infections and infestations	592	0.54(0.49,0.58)	0.55(0.51,0.60)	230.5017692	−0.86(−0.98)	0.55(0.51)
Eye disorders	358	0.87(0.78,0.96)	0.87(0.79,0.96)	7.013796167	−0.20(−0.35)	0.87(0.78)
Neoplasms benign, malignant and unspecified (incl cysts and polyps)	257	0.48(0.42,0.54)	0.49(0.43,0.55)	143.3942926	−1.04(−1.22)	0.49(0.43)
Surgical and medical procedures	240	0.85(0.75,0.97)	0.85(0.75,0.97)	6.039893685	−0.23(−0.41)	0.85(0.75)
Blood and lymphatic system disorders	210	0.60(0.53,0.69)	0.61(0.53,0.69)	54.46130333	−0.72(−0.92)	0.61(0.53)
Hepatobiliary disorders	190	1.01(0.87,1.16)	1.01(0.87,1.16)	0.006955001	0.01(−0.20)	1.01(0.87)
Immune system disorders	121	0.53(0.44,0.64)	0.53(0.45,0.64)	49.6531803	−0.90(−1.16)	0.53(0.45)
Ear and labyrinth disorders	98	1.11(0.91,1.35)	1.11(0.91,1.35)	1.03983678	0.15(−0.14)	1.11(0.91)
Endocrine disorders	76	1.45(1.16,1.81)	1.45(1.15,1.81)	10.44951326	0.53(0.19)	1.45(1.15)
Reproductive system and breast disorders	73	0.40(0.32,0.51)	0.41(0.32,0.51)	63.91872598	−1.30(−1.62)	0.41(0.32)
Congenital, familial and genetic disorders	61	1.01(0.78,1.29)	1.01(0.78,1.29)	0.002331756	0.01(−0.36)	1.01(0.78)
Social circumstances	27	0.28(0.19,0.41)	0.28(0.19,0.41)	49.97431	−1.83(−2.34)	0.28(0.19)
Product issues	24	0.07(0.05,0.10)	0.07(0.05,0.10)	301.0934965	−3.83(−4.36)	0.07(0.05)
Pregnancy, puerperium and perinatal conditions	18	0.21(0.13,0.33)	0.21(0.13,0.33)	54.42129429	−2.26(−2.86)	0.21(0.13)

Asterisks (*) indicate statistically significant signals. Abbreviations: ROR, reporting odds ratio; PRR, proportional reporting ratio; EBGM, empirical Bayesian geometric mean; EBGM05, the lower limit of the 95% confidence interval of EBGM; IC, information component; IC025, the lower limit of the 95% confidence interval of the IC.

**Table 4:** Signal strength of aliskiren related AEs across 28 SOCs in the VigiAccess database.

SOCs	Number of cases	ROR (95% CI)	PRR (95% CI)	chi-square	IC (IC025)	EBGM (EBGM05)
Investigations*	2495	2.54(2.43,2.65)	2.31(2.23,2.40)	1982.981347	1.21(1.15)	2.31(2.22)
General disorders and administration site conditions	2289	0.61(0.58,0.63)	0.66(0.64,0.69)	502.7642989	−0.60(−0.66)	0.66(0.63)
Nervous system disorders	1582	0.89(0.85,0.94)	0.90(0.86,0.95)	18.13400434	−0.15(−0.22)	0.90(0.86)
Gastrointestinal disorders	1582	0.93(0.88,0.97)	0.93(0.89,0.98)	8.639855324	−0.10(−0.18)	0.93(0.89)
Vascular disorders*	1129	3.25(3.06,3.45)	3.10(2.93,3.28)	1638.208225	1.63(1.54)	3.10(2.92)
Cardiac disorders*	1102	2.84(2.67,3.02)	2.72(2.57,2.88)	1225.367714	1.44(1.35)	2.72(2.56)
Skin and subcutaneous tissue disorders	1035	0.67(0.62,0.71)	0.69(0.65,0.73)	163.7881191	−0.54(−0.64)	0.69(0.64)
Respiratory, thoracic and mediastinal disorders	977	1.32(1.24,1.41)	1.30(1.22,1.38)	70.69486592	0.38(0.28)	1.30(1.22)
Renal and urinary disorders*	864	3.27(3.06,3.50)	3.16(2.96,3.37)	1294.389049	1.66(1.55)	3.16(2.95)
Metabolism and nutrition disorders*	701	2.35(2.18,2.53)	2.29(2.13,2.46)	519.9989158	1.20(1.08)	2.29(2.13)
Musculoskeletal and connective tissue disorders	660	0.72(0.67,0.78)	0.73(0.68,0.79)	67.51498094	−0.45(−0.56)	0.73(0.68)
Psychiatric disorders	508	0.62(0.56,0.67)	0.63(0.58,0.68)	118.7044836	−0.67(−0.80)	0.63(0.57)
Injury, poisoning and procedural complications	405	0.35(0.32,0.39)	0.37(0.34,0.41)	464.9936863	−1.44(−1.58)	0.37(0.34)
Infections and infestations	358	0.51(0.46,0.57)	0.52(0.47,0.58)	162.6902233	−0.94(−1.09)	0.52(0.47)
Eye disorders	330	1.12(1.00,1.25)	1.12(1.00,1.24)	4.195580942	0.16(0.00)	1.12(1.00)
Surgical and medical procedures	180	1.30(1.12,1.51)	1.30(1.12,1.50)	12.51101934	0.38(0.16)	1.30(1.12)
Neoplasms benign, malignant and unspecified (incl cysts and polyps)	161	0.67(0.58,0.78)	0.68(0.58,0.79)	25.51500288	−0.57(−0.79)	0.68(0.58)
Blood and lymphatic system disorders	143	0.37(0.32,0.44)	0.38(0.32,0.45)	149.0326454	−1.40(−1.64)	0.38(0.32)
Immune system disorders	110	0.51(0.42,0.61)	0.51(0.43,0.62)	51.77973069	−0.97(−1.23)	0.51(0.42)
Ear and labyrinth disorders	104	1.13(0.93,1.37)	1.13(0.93,1.37)	1.526412064	0.17(−0.11)	1.13(0.93)
Hepatobiliary disorders	99	0.73(0.60,0.88)	0.73(0.60,0.88)	10.25329041	−0.46(−0.75)	0.73(0.60)
Product issues	70	0.45(0.36,0.57)	0.45(0.36,0.57)	46.19058578	−1.14(−1.47)	0.45(0.36)
Reproductive system and breast disorders	61	0.33(0.26,0.43)	0.33(0.26,0.43)	81.61281851	−1.58(−1.93)	0.33(0.26)
Endocrine disorders	60	1.75(1.36,2.25)	1.75(1.36,2.25)	19.13368398	0.80(0.42)	1.75(1.35)
Congenital, familial and genetic disorders	47	1.62(1.22,2.16)	1.62(1.22,2.15)	11.08258671	0.69(0.26)	1.62(1.21)
Social circumstances	24	0.41(0.27,0.61)	0.41(0.27,0.61)	20.60842167	−1.29(−1.83)	0.41(0.27)
Pregnancy, puerperium and perinatal conditions	18	0.39(0.25,0.62)	0.39(0.25,0.62)	17.05580113	−1.35(−1.97)	0.39(0.25)

Asterisks (*) indicate statistically significant signals. Abbreviations: SOCs, system organ classes; AEs, adverse events; ROR, reporting odds ratio; PRR, proportional reporting ratio; EBGM, empirical Bayesian geometric mean; EBGM05, the lower limit of the 95% confidence interval of EBGM; IC, information component; IC025, the lower limit of the 95% confidence interval of the IC.

### Detection of AE signals at the PT level

At the PT level, the AE signals were analyzed using the reported cases and ROR algorithm. The top 30 AE detection results with the highest frequency and strongest signal intensity in the FAERS and VigiAccess databases were focused on. In the FAERS database, 2311 PTs related to aliskiren were detected. The top 30 PTs were listed in Table 5, sorted by the frequency of AE reports. The five most frequently reported PTs were blood pressure increased (n = 395), dyspnoea (n = 351), blood creatinine increased (n = 335), hypertension (n = 295), and dizziness (n = 287). The five PTs with the strongest AE signal intensity related to aliskiren were paradoxical pressor response (ROR(95% CI): 775.96(216.46–2781.64)), urine albumin/creatinine ratio increased (ROR(95% CI): 433.15(328.50–571.14)), and persistent cloaca (ROR(95% CI): 344.89(122.17–973.60)). The remaining PTs are detailed in Table 6. In the VigiAccess database, 2073 PTs related to aliskiren were identified. The top five PTs most frequently reported were blood pressure increased (n = 465), ineffective drug (n = 355), and dizziness (n = 334), hypertension (n = 314), and blood pressure increased (n = 287). The top 30 PTs in the VigiAccess database were sorted by the number of cases and signal intensity (as shown in Tables 5 and 6). In addition to the common adverse reactions explicitly mentioned in the instructions, we also discovered suspected adverse reactions not mentioned in the label, such as atrial fibrillation (n = 105), angina pectoris (n = 90), palpitations (n = 88), myalgia (n = 74), hypertensive crisis (n = 61), muscular weakness (n = 60), cardiac arrest (n = 53), pulmonary edema (n = 49), pollakiuria (n = 37), etc.

**Table 5:** Top 30 most frequent AEs for aliskiren at the preferred term (PT) level in FAERS and VigiAccess databases.

NO	FAERS database			VigiAccess database		
	PT	n	ROR (95% CI)	PT	n	ROR (95% CI)
1	Blood pressure increased*	395	7.94(7.18,8.77)	Blood pressure increased*	465	16.73(15.21,18.40)
2	Dyspnoea*	351	1.90(1.71,2.11)	Drug ineffective*	355	1.84(1.65,2.04)
3	Blood creatinine increased*	335	15.69(14.08,17.49)	Dizziness*	334	1.58(1.42,1.77)
4	Hypertension*	295	4.28(3.82,4.81)	Hypertension*	314	7.91(7.06,8.86)
5	Dizziness*	287	1.75(1.56,1.97)	Blood creatinine increased*	287	24.98(22.18,28.14)
6	Oedema peripheral*	228	5.61(4.93,6.40)	Diarrhoea*	277	1.64(1.45,1.85)
7	Diarrhoea	224	1.07(0.94,1.22)	Dyspnoea*	268	1.72(1.52,1.95)
8	Hypotension*	214	3.26(2.85,3.73)	Blood pressure inadequately controlled*	239	274.58(240.74,313.18)
9	Renal failure*	209	4.68(4.08,5.36)	Headache	226	0.71(0.62,0.81)
10	Headache	203	0.98(0.85,1.13)	Angioedema*	222	8.23(7.20,9.41)
11	Acute kidney injury*	197	3.06(2.66,3.52)	Oedema peripheral*	196	5.86(5.08,6.76)
12	Malaise*	189	1.29(1.12,1.49)	Hypotension*	194	3.93(3.41,4.54)
13	Drug ineffective	188	0.43(0.37,0.50)	Acute kidney injury*	178	5.05(4.35,5.86)
14	Cardiac failure*	187	7.11(6.16,8.22)	Nausea	177	0.53(0.46,0.62)
15	Blood pressure inadequately controlled*	185	93.62(80.82,108.46)	Fatigue	171	0.73(0.63,0.85)
16	Cerebrovascular accident*	184	3.26(2.82,3.77)	Hyperkalaemia*	171	23.73(20.38,27.64)
17	Nausea	183	0.70(0.61,0.81)	Rash	170	0.68(0.59,0.80)
18	Hyperkalaemia*	177	15.61(13.45,18.10)	Cough*	152	1.98(1.69,2.33)
19	Fatigue	176	0.69(0.59,0.80)	Cerebrovascular accident*	149	6.88(5.85,8.10)
20	Death	171	0.60(0.52,0.70)	Renal failure*	143	6.09(5.16,7.19)
21	Renal impairment*	160	5.90(5.05,6.90)	Malaise	141	1.14(0.97,1.35)
22	Angioedema*	155	10.12(8.64,11.85)	Pruritus	136	0.60(0.51,0.71)
23	Asthenia*	153	1.23(1.05,1.44)	Asthenia*	117	1.14(0.95,1.37)
24	Cough*	149	1.63(1.39,1.92)	Blood pressure decreased*	98	7.26(5.95,8.87)
25	Rash	148	0.99(0.84,1.17)	Palpitations*	95	1.80(1.47,2.20)
26	Vomiting	126	0.82(0.69,0.98)	Chest pain	93	1.15(0.94,1.41)
27	Chest pain*	123	2.00(1.67,2.38)	Death	93	0.82(0.67,1.00)
28	Diabetes mellitus*	121	4.76(3.98,5.70)	Renal impairment*	93	6.16(5.02,7.56)
29	Fall	118	1.08(0.90,1.29)	Swelling face*	91	6.15(5.00,7.57)
30	Pneumonia	116	1.03(0.86,1.24)	Feeling abnormal*	90	2.31(1.88,2.85)

Asterisks (*) indicate statistically significant signals. Abbreviations: AEs, adverse events; FAERS, FDA Adverse Event Reporting System; ROR, reporting odds ratio; PRR, proportional reporting ratio; EBGM, empirical Bayesian geometric mean; EBGM05, the lower limit of the 95% confidence interval of EBGM; IC, information component; IC025, the lower limit of the 95% confidence interval of the IC.

**Table 6:** Top 30 AEs with the highest signal intensity at the preferred term (PT) level in FAERS and VigiAccess databases.

NO	FAERS database			VigiAccess database		
	PT	n	ROR (95% CI)	PT	n	ROR (95% CI)
1	Paradoxical pressor response	3	775.96(216.46-2781.64)	Urine albumin/creatinine ratio increased	60	1232.49(936.12,1622.69)
2	Urine albumin/creatinine ratio increased	58	433.15(328.50-571.14)	Paradoxical pressor response	4	1199.97(414.94,3470.18)
3	Persistent cloaca	4	344.89(122.17-973.60)	Renin increased	24	750.18(492.16,1143.48)
4	Urachal abnormality	4	284.53(101.79-795.34)	Blood pressure inadequately controlled	239	219.24(192.59,249.59)
5	Renin increased	15	175.73(104.30-296.07)	Creatinine urine increased	11	188.41(103.49,343.02)
6	Conjoined twins	5	159.86(64.93-393.55)	Truncus arteriosus persistent	4	182.78(67.71,493.38)
7	Concomitant disease progression	89	139.26(112.52-172.35)	Albumin urine present	10	159.04(84.95,297.74)
8	Blood pressure inadequately controlled	185	93.62(80.82-108.46)	Percutaneous coronary intervention	8	151.26(75.06,304.82)
9	Congenital bladder anomaly	4	86.88(32.12-234.97)	Norepinephrine increased	3	147.84(47.11,464.02)
10	Microalbuminuria	20	79.43(50.92-123.89)	Microalbuminuria	19	126.16(80.13,198.66)
11	Primary hyperaldosteronism	4	77.95(28.87-210.51)	Blood aldosterone increased	4	101.47(37.81,272.34)
12	Angiocardiogram	4	77.42(28.67-209.06)	Concomitant disease progression	22	84.75(55.64,129.07)
13	Nephroangiosclerosis	4	74.88(27.74-202.10)	Withdrawal hypertension	3	81.49(26.10,254.38)
14	Blood aldosterone increased	4	70.69(26.21-190.66)	Blood pressure diastolic decreased	52	72.12(54.85,94.81)
15	Truncus arteriosus persistent	4	66.56(24.69-179.39)	Renal artery stenosis	15	65.96(39.66,109.71)
16	Creatinine urine increased	6	63.24(28.16-142.02)	Coronary angioplasty	5	64.25(26.63,155.01)
17	Neurologic neglect syndrome	10	60.68(32.43-113.55)	Therapy responder	3	53.90(17.31,167.88)
18	Percutaneous coronary intervention	5	52.31(21.59-126.69)	Congenital cardiovascular anomaly	5	50.15(20.80,120.88)
19	Renal artery stenosis	23	49.32(32.65-74.50)	Cardiac septal defect	5	49.22(20.42,118.63)
20	Albumin urine present	6	48.64(21.70-109.03)	Left ventricular hypertrophy	38	48.84(35.48,67.22)
21	Intestinal malrotation	4	44.98(16.75-120.80)	Defect conduction intraventricular	3	47.04(15.11,146.44)
22	Carotid artery thrombosis	11	44.22(24.37-80.23)	Organic erectile dysfunction	3	44.42(14.27,138.24)
23	Diabetic nephropathy	30	40.60(28.31-58.23)	Blood pressure systolic increased	84	40.70(32.83,50.47)
24	Coronary angioplasty	5	33.32(13.80-80.47)	Type II hypersensitivity	3	34.50(11.09,107.28)
25	Pulmonary vascular disorder	5	30.53(12.65-73.70)	Urine protein/creatinine ratio increased	3	34.10(10.97,106.04)
26	Dyspnoea paroxysmal nocturnal	7	27.74(13.18-58.41)	Glomerulosclerosis	3	30.85(9.92,95.90)
27	Adjustment disorder	10	24.92(13.37-46.45)	Hypertensive crisis	66	30.15(23.66,38.41)
28	Venous pressure jugular increased	3	24.39(7.83-75.99)	Electrocardiogram QRS complex prolonged	21	29.16(18.99,44.77)
29	Left ventricular hypertrophy	32	22.53(15.91-31.92)	Creatinine renal clearance increased	4	28.87(10.81,77.09)
30	Organic erectile dysfunction	3	20.82(6.69-64.82)	Diabetic nephropathy	13	27.16(15.75,46.84)

Abbreviations: AEs, adverse events; FAERS, FDA Adverse Event Reporting System; ROR, reporting odds ratio; PRR, proportional reporting ratio; EBGM, empirical Bayesian geometric mean; EBGM05, the lower limit of the 95% confidence interval of EBGM; IC, information component; IC025, the lower limit of the 95% confidence interval of the IC.

### Analysis of the onset time of aliskiren AEs

**Fig 5:**
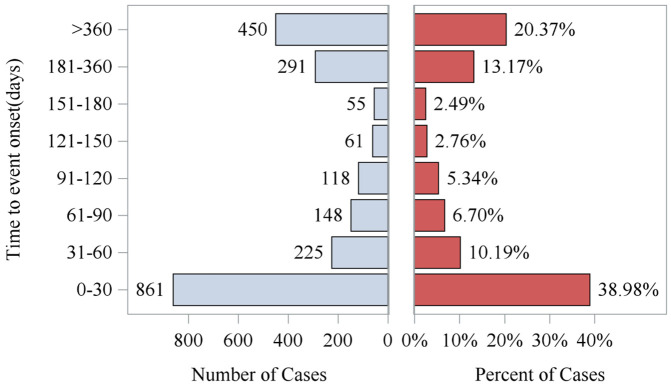
Time to onset of adverse events induced by aliskiren. Distribution of adverse event onset time intervals (days) among study reports, presented as both the number of cases and corresponding percentage of total cases for each interval.

**Fig 6:**
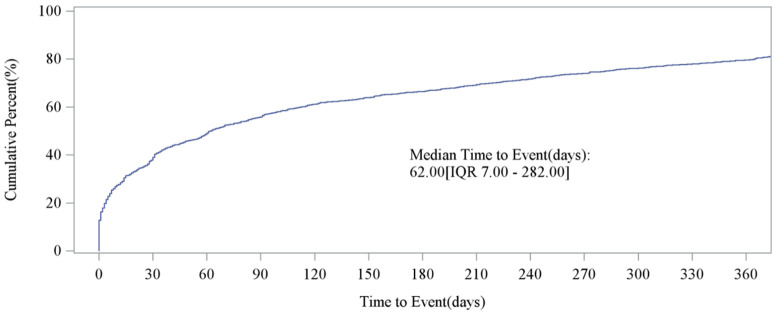
Cumulative incidence of adverse events related to aliskiren, highlighting the early risk period. Distribution of time to adverse event onset (days) for the aliskiren, with a median time to event of 62.00 days and an interquartile range (IQR) of 7.00-282.00 days.

After excluding the reports that were not reported or had incorrect start times, a total of 2209 reports met the inclusion criteria. The analysis of the time of AEs during aliskiren treatment for primary hypertension showed that most AEs occurred within the first 30 days of treatment (861 cases, accounting for 38.98%) and after 360 days of medication (a total of 450 cases, accounting for 20.37%) (Fig 5). The cumulative incidence curve of AEs for aliskiren is shown in Fig 6. The results indicated that the median time to onset (TTO) of AEs was 62 days (interquartile range (IQR): 7–282 days).

## Discussion

Both the FAERS and VigiAccess databases collect reports of suspected adverse reactions through the spontaneous reporting system (SRS), with the aim of continuously monitoring the safety of drugs and vaccines by collecting and analyzing voluntarily submitted reports of adverse drug reactions [19]. The data from FAERS database mainly comes from medical institutions, consumers, and pharmaceutical companies in the United States (as mandated by law), and is updated quarterly. The data from VigiAccess database comes from drug regulatory agencies in 130 + countries worldwide and is updated regularly (the specific frequency is not disclosed). Therefore, the two databases have overlapping and complementary features. In this study, the integrated application of the FAERS and VigiAccess databases demonstrated how data complementarity, methodological synergy, and global perspective integration significantly enhanced the efficacy of pharmacovigilance. This collaborative approach accelerates the speed of risk identification and verification, and establishes a scientific foundation for personalized medicine and global regulatory supervision.

Aliskiren is one of the oral drugs for treating primary hypertension. In a multicenter, randomized, double-blind, placebo-controlled parallel-group study, 652 patients with mild to moderate hypertension were randomly divided into the placebo group, the 150-mg aliskiren group, the 300-mg aliskiren group, the 600-mg aliskiren group (with good tolerability), and the 150-mg irbesartan group for a double-blind treatment trial [20]. The results showed that compared with the patients in the placebo group, the patients in the aliskiren group had significantly lower blood pressure (BP). There was no significant difference in the antihypertensive effect between 150-mg aliskiren and irbesartan, while the antihypertensive effect of 300-mg and 600-mg aliskiren was significantly better than that of irbesartan [20]. The most common adverse reactions were headache, dizziness, and diarrhea. The incidence of headache was 2.4%, 6.2%, and 4.6% when using 150-mg, 300-mg, and 600-mg aliskiren, respectively, while the proportion in the placebo group was 5.3% [20].

Aliskiren has also been proven to be effective in reducing central arterial pressure (CAP) when used in combination with other antihypertensive drugs [21]. The Aliskiren for Geriatric Lowering of SyStolic hypertension (AGELESS) study investigated the efficacy and safety of aliskiren versus ramipril in treating elderly patients with primary systolic hypertension [22]. In this study, 901 elderly hypertensive patients participated in a 36-week randomized, double-blind, parallel-group, active-controlled, optional add-on therapy trial [23]. The results showed that the proportion of patients requiring the combination of other drugs was lower in the aliskiren-based treatment group [23]; moreover, the proportion of adverse reactions such as cough was also lower in the aliskiren group than in the ramipril group [23]. The efficacy of aliskiren in treating elderly systolic hypertension was superior to that of ramipril.

Aliskiren has significant effects in lowering BP, improving cardiac function, and protecting renal function [24,25]. However, some side effects also occur when this drug is used in treatment. Uresin and colleagues tested the efficacy, safety, and tolerance of the combination therapy of aliskiren and ACEI/ARB [26]. They found that there was no statistically significant difference in tolerance and safety among the treatment groups [26]. The most common AEs of aliskiren monotherapy were headache (3.2%), cough (2.1%), nasopharyngitis (3.2%), and diarrhea (1.1%), while the rate of significantly elevated serum creatinine levels was relatively low [26]. In the The Aliskiren Trial in Type 2 Diabetes Using Cardio-renal Disease Endpoints (ALTITUDE) trial, patients with diabetes and renal dysfunction who were treated with aliskiren and ACEI/ARB were prematurely terminated due to an increase in AEs (including non-fatal strokes, renal dysfunction, hyperkalemia, and hypotension) [27]. Based on this study, it is not recommended to combine aliskiren and ACEI/ARB for the treatment of patients with hypertension and diabetes or at least moderate renal dysfunction [27,28].

Several large multicenter studies have reported the common AEs of aliskiren. However, some new and serious AEs that occurred after the drug was launched have not been reported in detail. Through the use of real-world data from the FAERS and VigiAccess databases, this study comprehensively evaluated the AEs after the launch of aliskiren. Our analysis showed that the frequency of AEs reported by female patients was higher than that of male patients in both the FAERS and VigiAccess databases. The majority of AE reports were from patients aged 65 and above. Considering that the physiological decline in renal function in the elderly, the sluggishness of the baroreceptor reflex, and the poor regulation of the autonomic nervous system make them more sensitive to antihypertensive drugs [29,30]. And most elderly people have multiple chronic diseases that require multiple medications, which accelerates the occurrence of AEs. Aliskiren inhibits renin at the source, and its thorough action combined with the unique metabolic characteristics of the elderly led to an increase in AEs. Therefore, when using aliskiren for the elderly, it is advisable to avoid using it in combination with ACEI or ARB. In patients with diabetes, the combination is prohibited [27]. Start with a low dose and increase gradually; regularly monitor BP, blood potassium, and renal function, etc. Most aliskiren-related AEs occurred within the first 30 days of treatment and after 360 days of medication. Therefore, it is necessary to remind doctors to strengthen monitoring of patients using this drug during these two periods to avoid serious consequences. Patients, as the parties involved, should cooperate with the doctor and follow the long-term follow-up and blood test plans set by physicians. This is an effective method to detect “asymptomatic but dangerous” issues (such as slowly rising blood potassium).

The most common serious outcomes reported in the aliskiren AE reports are hospitalization and death. Through systematic data mining, this study confirmed all the AEs listed in the drug label, and aimed to identify unreported adverse reactions related to aliskiren, with a particular focus on detecting new and rare AEs. These findings provide evidence for the prevention and management of these AEs.

In the FAERS database, some AEs related to aliskiren, although rarely reported, such as glomerular sclerosis, ischemic cerebral infarction, vestibular neuritis, increased jugular venous pressure, gouty arthritis, cerebellar hemorrhage, calcium metabolism disorders, and cardiogenic asthma, may have potential clinical significance. These findings provide clinicians with comprehensive risk management insights to enhance patient safety monitoring. In the VigiAccess database, rare but severe AEs related to aliskiren were identified, including duodenal tumors, acute right ventricular failure, malignant urinary tract tumors, ventricular tachyarrhythmias, epidermolysis bullosa acquisita, malignant hypertension, and lung tumors. These findings highlight the need for regular cardiovascular and cerebrovascular examinations, as well as tumor marker tests, in patients using aliskiren. It is worth noting that dizziness, headache, diarrhea, fatigue, cough, rash, and hyperkalemia were all highly prevalent in both the FAERS and VigiAccess databases, which is consistent with the results of previous clinical trials. These adverse reactions are mainly related to the mechanism of action and metabolism of aliskiren (Fig 1).

## Conclusion

This comprehensive retrospective pharmacovigilance study, utilizing data from the FAERS and VigiAccess databases, provides a large-scale, real-world safety profile of aliskiren. Our analysis confirms its established safety concerns, such as renal impairment, hyperkalemia, hypotension, and dizziness, while also identifying potential signals for new and rare AEs.

Beyond the labeled AEs, our data mining revealed notable signals for conditions not prominently featured in the prescribing information, including atrial fibrillation, angina pectoris, hypertensive crisis, and muscular weakness. Additionally, while reported infrequently, signals for serious and rare events such as paradoxical pressor response, ischemic stroke, and certain malignancies warrant further clinical attention and investigation. Analysis of the onset time revealed that AEs presented a bimodal distribution. This pattern highlights two critical periods that require heightened vigilance: the initial stage of acute reactions (such as hypotension and hyperkalemia) and the long-term stage (such as chronic renal changes and electrolyte imbalances). In summary, this study validates the known safety profile of aliskiren from clinical trials within a real-world context and expands the understanding of its potential risks. These insights offer actionable guidance for clinicians to optimize the safe use of aliskiren, ultimately aiming to improve patient outcomes in hypertension management

## Supporting information

S1 Table Two-by-two contingency table. Two-by-two contingency table for disproportionality analyses. (DOCX)

S2 Table Four major algorithms. Four major algorithms used for signal detection. (DOCX)

S3 Table FAERS Database Mining Report. (DOCX)

S4 Table MedDRA hierarchical signal analysis-Vigiaccess database. (XLSX)
