# Supplementary material for: Real-world safety of aliskiren in primary hypertension: A cross-database study
Source: PLoS One. 2026 Apr 3;21(4):e0346326. doi: 10.1371/journal.pone.0346326 (PMC13048407; doi:10.1371/journal.pone.0346326)
Supplement: S1 Table — (DOCX) [file pone.0346326.s001.docx]

**Supplementary Table 1**: Two-by-two contingency table for disproportionality analyses.

| **Item** | **Target AEs** | **Other AEs** | **Total** |
| --- | --- | --- | --- |
| Target drug | a | b | a+b |
| Other drugs | c | d | c+d |
| Total | a+c | b+d | a+b+c+d |

Abbreviation: AEs, adverse events; a, number of reports containing both the target drug and target adverse drug reaction; b, number of reports containing other adverse drug reaction of the target drug; c, number of reports containing the target adverse drug reaction of other drugs; d, number of reports containing other drugs and other adverse drug reactions.
