# Supplementary material for: Real-world safety of aliskiren in primary hypertension: A cross-database study
Source: PLoS One. 2026 Apr 3;21(4):e0346326. doi: 10.1371/journal.pone.0346326 (PMC13048407; doi:10.1371/journal.pone.0346326)
Supplement: S2 Table — (DOCX) [file pone.0346326.s002.docx]

**Supplementary Table 2**: Four major algorithms used for signal detection.

| **Algorithms** | **Equation** | **Criteria** |
| --- | --- | --- |
| ROR | ROR=ad/b/c | lower limit of 95% CI>1, N≥3 |
|  | 95%CI=eln(ROR)±1.96(1/a+1/b+1/c+1/d)^0.5 |  |
| PRR | PRR=a(c+d)/c/(a+b) | PRR≥2, χ2≥4, N≥3 |
|  | χ2=[(ad-bc)^2](a+b+c+d)/[(a+b)(c+d)(a+c)(b+d)] |  |
| BCPNN | IC=log2a(a+b+c+d)(a+c)(a+b) | IC025>0 |
|  | 95%CI= E(IC) ± 2V(IC)^0.5 |  |
| MGPS | EBGM=a(a+b+c+d)/(a+c)/(a+b) | EBGM05>2 |
|  | 95%CI=eln(EBGM)±1.96(1/a+1/b+1/c+1/d)^0.5 |  |

Abbreviation: a, number of reports containing both the target drug and target adverse drug reaction; b, number of reports containing other adverse drug reaction of the target drug; c, number of reports containing the target adverse drug reaction of other drugs; d, number of reports containing other drugs and other adverse drug reactions. 95%CI, 95% confidence interval; N, the number of reports; χ2, chi-squared; IC, information component; IC025, the lower limit of 95% CI of the IC; E(IC), the IC expectations; V(IC), the variance of IC; EBGM, empirical Bayesian geometric mean; EBGM05, the lower limit of 95% CI of EBGM.
