# Supplementary material for: Real-world safety of aliskiren in primary hypertension: A cross-database study
Source: PLoS One. 2026 Apr 3;21(4):e0346326. doi: 10.1371/journal.pone.0346326 (PMC13048407; doi:10.1371/journal.pone.0346326)
Supplement: S3 Table — (DOCX) [file pone.0346326.s003.docx]

TABLE 1 Characteristics of AEs reports

| **Characteristics** | **n (%)** |
| --- | --- |
| Sex |  |
| Female | 2701(48.27) |
| Male | 2430(43.42) |
| Not Specified | 465( 8.31) |
| Age(years) |  |
| <18 | 6( 0.11) |
| 18-44 | 139( 2.48) |
| 45-64 | 944(16.87) |
| ≥65 | 1747(31.22) |
| NotSpecified | 2760(49.32) |
| Age(years) |  |
| N(Missing) | 2836(2760) |
| Mean(SD) | 67.27(13.15) |
| Median(Q1,Q3) | 69.00(60.00,77.00) |
| Min,Max | 0.00,101.00 |
| Report year |  |
| 2005 | 1( 0.02) |
| 2007 | 616(11.01) |
| 2008 | 819(14.64) |
| 2009 | 506( 9.04) |
| 2010 | 773(13.81) |
| 2011 | 742(13.26) |
| 2012 | 798(14.26) |
| 2013 | 407( 7.27) |
| 2014 | 172( 3.07) |
| 2015 | 243( 4.34) |
| 2016 | 188( 3.36) |
| 2017 | 86( 1.54) |
| 2018 | 94( 1.68) |
| 2019 | 61( 1.09) |
| 2020 | 48( 0.86) |
| 2021 | 11( 0.20) |
| 2022 | 10( 0.18) |
| 2023 | 10( 0.18) |
| 2024 | 6( 0.11) |
| 2025 | 5( 0.09) |
| Reporter |  |
| Consumer | 1713(30.61) |
| Lawyer | 24( 0.43) |
| Not Specified | 176( 3.15) |
| Other health-professional | 517( 9.24) |
| Pharmacist | 247( 4.41) |
| Physician | 2919(52.16) |
| Outcomes |  |
| Life-Threatening | 277( 4.95) |
| Hospitalization - Initial or Prolonged | 1735(31.00) |
| Disability | 98( 1.75) |
| Death | 548( 9.79) |
| Congenital Anomaly | 8( 0.14) |
| Required Intervention to Prevent Permanent Impairment/Damage | 4( 0.07) |
| Other | 3080(55.04) |
| Adverse event occurrence time(days) |  |
| 0-30d | 861(15.39) |
| 31-60d | 225( 4.02) |
| 61-90d | 148( 2.64) |
| 91-120d | 118( 2.11) |
| 121-150d | 61( 1.09) |
| 151-180d | 55( 0.98) |
| 181-360d | 291( 5.20) |
| ＞360d | 450( 8.04) |
| Missing or outlier(less than 0)(%) | 3387(60.53) |
| Adverse event occurrence time(days) |  |
| N(Missing) | 2209(3387) |
| Mean(SD) | 218.85(391.36) |
| Median(Q1,Q3) | 62.00(7.00,282.00) |
| Min,Max | 0.00,9133.00 |
| Weight(kg) |  |
| N(Missing) | 1644(3952) |
| Mean(SD) | 80.49(23.03) |
| Median(Q1,Q3) | 78.00(65.00,92.00) |
| Min,Max | 0.06,249.80 |

TABLE 2 Signal strength of ADEs at the System Organ Class (SOC) level in FAERS database

| **System Organ Class(SOC)** | **SOC Code** | **Case reports** | **ROR(95% CI)** | **PRR(95% CI)** | **Chi_Square** | **IC(IC025)** | **EBGM(EBGM05)** |
| --- | --- | --- | --- | --- | --- | --- | --- |
| Investigations | 10022891 | 2773 | 2.42(2.32,2.52) | 2.22(2.15,2.30) | 1989.46 | 1.15(1.09) | 2.22(2.14) |
| General disorders and administration site conditions | 10018065 | 2651 | 0.70(0.68,0.73) | 0.74(0.72,0.77) | 285.09 | -0.43(-0.49) | 0.74(0.71) |
| Nervous system disorders | 10029205 | 1836 | 1.07(1.02,1.13) | 1.07(1.02,1.11) | 8.19 | 0.09(0.02) | 1.07(1.02) |
| Gastrointestinal disorders | 10017947 | 1672 | 0.96(0.91,1.01) | 0.96(0.92,1.01) | 2.88 | -0.06(-0.13) | 0.96(0.91) |
| Cardiac disorders | 10007541 | 1655 | 3.31(3.15,3.48) | 3.12(2.98,3.27) | 2451.56 | 1.64(1.57) | 3.12(2.97) |
| Respiratory, thoracic and mediastinal disorders | 10038738 | 1318 | 1.40(1.32,1.48) | 1.37(1.30,1.44) | 138.87 | 0.46(0.37) | 1.37(1.30) |
| Renal and urinary disorders | 10038359 | 1217 | 3.30(3.12,3.50) | 3.16(3.00,3.34) | 1834.19 | 1.66(1.57) | 3.16(2.98) |
| Vascular disorders | 10047065 | 1169 | 2.80(2.64,2.97) | 2.70(2.55,2.85) | 1274.02 | 1.43(1.34) | 2.70(2.54) |
| Skin and subcutaneous tissue disorders | 10040785 | 963 | 0.86(0.81,0.92) | 0.87(0.81,0.92) | 21.20 | -0.21(-0.30) | 0.87(0.81) |
| Metabolism and nutrition disorders | 10027433 | 905 | 2.09(1.96,2.24) | 2.04(1.92,2.18) | 492.19 | 1.03(0.93) | 2.04(1.91) |
| Musculoskeletal and connective tissue disorders | 10028395 | 738 | 0.69(0.64,0.74) | 0.70(0.65,0.75) | 99.22 | -0.51(-0.62) | 0.70(0.65) |
| Injury, poisoning and procedural complications | 10022117 | 623 | 0.27(0.25,0.29) | 0.29(0.27,0.31) | 1227.32 | -1.80(-1.91) | 0.29(0.27) |
| Psychiatric disorders | 10037175 | 596 | 0.51(0.47,0.55) | 0.52(0.48,0.57) | 273.22 | -0.93(-1.05) | 0.52(0.48) |
| Infections and infestations | 10021881 | 592 | 0.54(0.49,0.58) | 0.55(0.51,0.60) | 230.50 | -0.86(-0.98) | 0.55(0.51) |
| Eye disorders | 10015919 | 358 | 0.87(0.78,0.96) | 0.87(0.79,0.96) | 7.01 | -0.20(-0.35) | 0.87(0.78) |
| Neoplasms benign, malignant and unspecified (incl cysts and polyps) | 10029104 | 257 | 0.48(0.42,0.54) | 0.49(0.43,0.55) | 143.39 | -1.04(-1.22) | 0.49(0.43) |
| Surgical and medical procedures | 10042613 | 240 | 0.85(0.75,0.97) | 0.85(0.75,0.97) | 6.04 | -0.23(-0.41) | 0.85(0.75) |
| Blood and lymphatic system disorders | 10005329 | 210 | 0.60(0.53,0.69) | 0.61(0.53,0.69) | 54.46 | -0.72(-0.92) | 0.61(0.53) |
| Hepatobiliary disorders | 10019805 | 190 | 1.01(0.87,1.16) | 1.01(0.87,1.16) | 0.01 | 0.01(-0.20) | 1.01(0.87) |
| Immune system disorders | 10021428 | 121 | 0.53(0.44,0.64) | 0.53(0.45,0.64) | 49.65 | -0.90(-1.16) | 0.53(0.45) |
| Ear and labyrinth disorders | 10013993 | 98 | 1.11(0.91,1.35) | 1.11(0.91,1.35) | 1.04 | 0.15(-0.14) | 1.11(0.91) |
| Endocrine disorders | 10014698 | 76 | 1.45(1.16,1.81) | 1.45(1.15,1.81) | 10.45 | 0.53(0.19) | 1.45(1.15) |
| Reproductive system and breast disorders | 10038604 | 73 | 0.40(0.32,0.51) | 0.41(0.32,0.51) | 63.92 | -1.30(-1.62) | 0.41(0.32) |
| Congenital, familial and genetic disorders | 10010331 | 61 | 1.01(0.78,1.29) | 1.01(0.78,1.29) | 0.00 | 0.01(-0.36) | 1.01(0.78) |
| Social circumstances | 10041244 | 27 | 0.28(0.19,0.41) | 0.28(0.19,0.41) | 49.97 | -1.83(-2.34) | 0.28(0.19) |
| Product issues | 10077536 | 24 | 0.07(0.05,0.10) | 0.07(0.05,0.10) | 301.09 | -3.83(-4.36) | 0.07(0.05) |
| Pregnancy, puerperium and perinatal conditions | 10036585 | 18 | 0.21(0.13,0.33) | 0.21(0.13,0.33) | 54.42 | -2.26(-2.86) | 0.21(0.13) |

Note:ranked by case reports

TABLE 3 Signal strength of adverse events at the Preferred Term(PT) level ranked by Reports

| **System Organ Class(SOC)** | **Preferred Term(PT)** | **Case reports** | **ROR (95% CI)** | **PRR (95% CI)** | **Chi Square** | **IC (IC025)** | **EBGM (EBGM05)** |
| --- | --- | --- | --- | --- | --- | --- | --- |
| Investigations | Blood pressure increased | 395 | 7.94  (7.18,8.77) | 7.80  (7.08,8.60) | 2342.27 | 2.96  (2.79) | 7.78  (7.05) |
| Investigations | Blood creatinine increased | 335 | 15.69  (14.08,17.49) | 15.45  (13.89,17.19) | 4508.81 | 3.94  (3.72) | 15.38  (13.80) |
| Vascular disorders | Hypertension | 295 | 4.28  (3.82,4.81) | 4.24  (3.78,4.75) | 731.08 | 2.08  (1.90) | 4.23  (3.77) |
| General disorders and administration site conditions | Oedema peripheral | 228 | 5.61  (4.93,6.40) | 5.56  (4.89,6.33) | 853.18 | 2.47  (2.25) | 5.55  (4.87) |
| Vascular disorders | Hypotension | 214 | 3.26  (2.85,3.73) | 3.24  (2.83,3.70) | 331.80 | 1.69  (1.48) | 3.24  (2.83) |
| Renal and urinary disorders | Renal failure | 209 | 4.68  (4.08,5.36) | 4.64  (4.06,5.31) | 597.58 | 2.21  (1.99) | 4.64  (4.05) |
| Renal and urinary disorders | Acute kidney injury | 197 | 3.06  (2.66,3.52) | 3.04  (2.65,3.50) | 270.80 | 1.60  (1.38) | 3.04  (2.64) |
| Cardiac disorders | Cardiac failure | 187 | 7.11  (6.16,8.22) | 7.06  (6.12,8.14) | 970.87 | 2.82  (2.56) | 7.04  (6.10) |
| Vascular disorders | Blood pressure inadequately controlled | 185 | 93.62  (80.82,108.46) | 92.79  (80.20,107.35) | 16268.4 | 6.49  (5.71) | 89.89  (77.59) |
| Nervous system disorders | Cerebrovascular accident | 184 | 3.26  (2.82,3.77) | 3.24  (2.80,3.74) | 285.00 | 1.69  (1.46) | 3.23  (2.80) |
| Metabolism and nutrition disorders | Hyperkalaemia | 177 | 15.61  (13.45,18.10) | 15.48  (13.36,17.93) | 2385.53 | 3.94  (3.61) | 15.40  (13.28) |
| Renal and urinary disorders | Renal impairment | 160 | 5.90  (5.05,6.90) | 5.87  (5.03,6.85) | 645.36 | 2.55  (2.28) | 5.86  (5.01) |
| Skin and subcutaneous tissue disorders | Angioedema | 155 | 10.12  (8.64,11.85) | 10.05  (8.59,11.76) | 1259.41 | 3.32  (3.01) | 10.02  (8.55) |
| Metabolism and nutrition disorders | Diabetes mellitus | 121 | 4.76  (3.98,5.70) | 4.74  (3.97,5.66) | 357.03 | 2.24  (1.94) | 4.73  (3.96) |
| Investigations | Blood urea increased | 111 | 19.56  (16.22,23.59) | 19.46  (16.16,23.44) | 1931.30 | 4.27  (3.78) | 19.34  (16.04) |
| Nervous system disorders | Syncope | 109 | 3.26  (2.70,3.94) | 3.25  (2.70,3.92) | 169.98 | 1.70  (1.39) | 3.25  (2.69) |
| Cardiac disorders | Atrial fibrillation | 105 | 3.27  (2.70,3.96) | 3.25  (2.69,3.94) | 164.03 | 1.70  (1.39) | 3.25  (2.68) |
| Renal and urinary disorders | Chronic kidney disease | 102 | 3.61  (2.97,4.38) | 3.59  (2.96,4.36) | 190.81 | 1.84  (1.52) | 3.59  (2.95) |
| Investigations | Blood pressure decreased | 96 | 4.39  (3.59,5.36) | 4.37  (3.58,5.34) | 249.50 | 2.13  (1.78) | 4.37  (3.57) |
| Cardiac disorders | Angina pectoris | 90 | 9.34  (7.59,11.49) | 9.30  (7.57,11.44) | 665.18 | 3.21  (2.78) | 9.28  (7.54) |
| General disorders and administration site conditions | Concomitant disease progression | 89 | 139.26  (112.52,172.35) | 138.65  (112.13,171.45) | 11598.0 | 7.05  (5.44) | 132.26  (106.86) |
| Investigations | Blood potassium increased | 86 | 16.14  (13.05,19.96) | 16.08  (13.01,19.86) | 1209.49 | 4.00  (3.46) | 15.99  (12.93) |
| Metabolism and nutrition disorders | Hyponatraemia | 83 | 4.47  (3.60,5.55) | 4.46  (3.60,5.53) | 222.41 | 2.15  (1.78) | 4.45  (3.59) |
| Cardiac disorders | Arrhythmia | 82 | 5.18  (4.17,6.44) | 5.17  (4.16,6.41) | 275.32 | 2.37  (1.98) | 5.16  (4.15) |
| General disorders and administration site conditions | Oedema | 82 | 4.60  (3.70,5.72) | 4.59  (3.70,5.69) | 229.86 | 2.20  (1.82) | 4.58  (3.69) |
| General disorders and administration site conditions | Swelling face | 77 | 3.60  (2.88,4.50) | 3.59  (2.87,4.49) | 143.75 | 1.84  (1.47) | 3.59  (2.87) |
| Investigations | Blood pressure systolic increased | 73 | 11.39  (9.05,14.34) | 11.35  (9.02,14.28) | 686.54 | 3.50  (2.97) | 11.31  (8.98) |
| Investigations | Glycosylated haemoglobin increased | 70 | 8.46  (6.69,10.70) | 8.44  (6.67,10.66) | 457.65 | 3.07  (2.59) | 8.41  (6.65) |
| Renal and urinary disorders | Proteinuria | 62 | 10.26  (7.99,13.17) | 10.23  (7.98,13.13) | 514.84 | 3.35  (2.79) | 10.20  (7.95) |
| Cardiac disorders | Bradycardia | 61 | 3.44  (2.68,4.43) | 3.44  (2.67,4.41) | 105.30 | 1.78  (1.36) | 3.43  (2.67) |
| Vascular disorders | Hypertensive crisis | 61 | 16.62  (12.92,21.39) | 16.57  (12.89,21.31) | 887.69 | 4.04  (3.35) | 16.48  (12.81) |
| Investigations | Glomerular filtration rate decreased | 58 | 15.14  (11.69,19.61) | 15.10  (11.67,19.54) | 759.90 | 3.91  (3.22) | 15.03  (11.61) |
| Investigations | Urine albumin/creatinine ratio increased | 58 | 433.15  (328.50,571.14) | 431.93  (327.79,569.14) | 21649.0 | 8.55  (5.27) | 375.12  (284.49) |
| Renal and urinary disorders | Renal disorder | 57 | 3.70  (2.86,4.80) | 3.70  (2.85,4.79) | 112.03 | 1.88  (1.44) | 3.69  (2.85) |
| Cardiac disorders | Coronary artery disease | 53 | 5.04  (3.85,6.60) | 5.03  (3.84,6.58) | 170.82 | 2.33  (1.83) | 5.02  (3.83) |
| Gastrointestinal disorders | Lip swelling | 53 | 4.85  (3.70,6.35) | 4.84  (3.70,6.34) | 161.30 | 2.27  (1.78) | 4.83  (3.69) |
| Gastrointestinal disorders | Swollen tongue | 52 | 5.04  (3.83,6.61) | 5.03  (3.83,6.59) | 167.45 | 2.33  (1.82) | 5.02  (3.82) |
| Respiratory, thoracic and mediastinal disorders | Pulmonary oedema | 49 | 3.29  (2.48,4.35) | 3.28  (2.48,4.34) | 77.62 | 1.71  (1.24) | 3.28  (2.48) |
| Investigations | Blood pressure diastolic decreased | 48 | 19.65  (14.79,26.11) | 19.60  (14.76,26.03) | 841.79 | 4.28  (3.41) | 19.48  (14.66) |
| Vascular disorders | Blood pressure fluctuation | 47 | 5.89  (4.43,7.85) | 5.88  (4.42,7.83) | 190.20 | 2.55  (2.00) | 5.87  (4.41) |
| Respiratory, thoracic and mediastinal disorders | Pharyngeal oedema | 41 | 7.68  (5.65,10.44) | 7.67  (5.65,10.42) | 237.25 | 2.94  (2.28) | 7.65  (5.63) |
| Nervous system disorders | Dysarthria | 40 | 3.21  (2.35,4.37) | 3.20  (2.35,4.36) | 60.51 | 1.68  (1.15) | 3.20  (2.35) |
| Investigations | Heart rate decreased | 40 | 3.41  (2.50,4.65) | 3.40  (2.50,4.64) | 67.78 | 1.76  (1.23) | 3.40  (2.49) |
| General disorders and administration site conditions | Face oedema | 35 | 6.20  (4.45,8.64) | 6.19  (4.45,8.63) | 152.09 | 2.63  (1.95) | 6.18  (4.44) |
| Cardiac disorders | Acute myocardial infarction | 34 | 3.39  (2.42,4.75) | 3.39  (2.42,4.74) | 57.23 | 1.76  (1.18) | 3.39  (2.42) |
| Nervous system disorders | Transient ischaemic attack | 34 | 2.99  (2.14,4.19) | 2.99  (2.13,4.18) | 44.91 | 1.58  (1.01) | 2.98  (2.13) |
| Nervous system disorders | Cerebral infarction | 32 | 3.92  (2.77,5.54) | 3.91  (2.77,5.53) | 69.34 | 1.97  (1.34) | 3.91  (2.76) |
| Investigations | Gamma-glutamyltransferase increased | 32 | 4.29  (3.03,6.07) | 4.29  (3.03,6.06) | 80.54 | 2.10  (1.46) | 4.28  (3.03) |
| Cardiac disorders | Left ventricular hypertrophy | 32 | 22.53  (15.91,31.92) | 22.50  (15.89,31.85) | 652.29 | 4.48  (3.26) | 22.33  (15.77) |
| Renal and urinary disorders | Diabetic nephropathy | 30 | 40.60  (28.31,58.23) | 40.54  (28.28,58.12) | 1140.81 | 5.32  (3.62) | 39.99  (27.88) |
| Cardiac disorders | Ventricular tachycardia | 29 | 5.29  (3.68,7.62) | 5.29  (3.67,7.61) | 100.61 | 2.40  (1.68) | 5.28  (3.67) |
| Cardiac disorders | Cardiomegaly | 28 | 6.58  (4.54,9.53) | 6.57  (4.53,9.51) | 131.90 | 2.71  (1.92) | 6.56  (4.52) |
| Cardiac disorders | Extrasystoles | 27 | 11.51  (7.88,16.79) | 11.49  (7.88,16.77) | 257.61 | 3.52  (2.51) | 11.45  (7.84) |
| Cardiac disorders | Myocardial ischaemia | 27 | 6.89  (4.72,10.06) | 6.89  (4.72,10.04) | 135.53 | 2.78  (1.96) | 6.87  (4.71) |
| Investigations | Urine output decreased | 27 | 9.21  (6.31,13.44) | 9.20  (6.31,13.42) | 196.66 | 3.20  (2.28) | 9.17  (6.28) |
| Gastrointestinal disorders | Gastritis | 26 | 2.95  (2.01,4.33) | 2.95  (2.01,4.33) | 33.43 | 1.56  (0.90) | 2.95  (2.00) |
| Nervous system disorders | Hemiparesis | 26 | 4.58  (3.12,6.73) | 4.58  (3.12,6.72) | 72.56 | 2.19  (1.46) | 4.57  (3.11) |
| Eye disorders | Periorbital oedema | 26 | 15.31  (10.41,22.52) | 15.29  (10.41,22.48) | 345.53 | 3.93  (2.76) | 15.22  (10.35) |
| Investigations | Haematocrit decreased | 25 | 3.71  (2.50,5.49) | 3.70  (2.50,5.48) | 49.27 | 1.89  (1.18) | 3.70  (2.50) |
| Eye disorders | Eyelid oedema | 24 | 6.05  (4.05,9.03) | 6.04  (4.05,9.02) | 100.85 | 2.59  (1.75) | 6.03  (4.04) |
| Nervous system disorders | Hemiplegia | 24 | 8.65  (5.79,12.91) | 8.64  (5.79,12.89) | 161.62 | 3.11  (2.14) | 8.61  (5.77) |
| Cardiac disorders | Mitral valve incompetence | 24 | 6.32  (4.23,9.43) | 6.31  (4.23,9.42) | 107.04 | 2.66  (1.80) | 6.30  (4.22) |
| Investigations | Blood uric acid increased | 23 | 12.23  (8.12,18.43) | 12.22  (8.12,18.40) | 235.97 | 3.61  (2.46) | 12.17  (8.08) |
| Cardiac disorders | Cardiac failure acute | 23 | 10.62  (7.05,16.00) | 10.61  (7.05,15.97) | 199.47 | 3.40  (2.33) | 10.57  (7.02) |
| General disorders and administration site conditions | Generalised oedema | 23 | 5.75  (3.82,8.66) | 5.74  (3.82,8.64) | 89.94 | 2.52  (1.67) | 5.73  (3.81) |
| Renal and urinary disorders | Renal artery stenosis | 23 | 49.32  (32.65,74.50) | 49.27  (32.63,74.39) | 1069.16 | 5.60  (3.43) | 48.45  (32.07) |
| Respiratory, thoracic and mediastinal disorders | Sleep apnoea syndrome | 23 | 3.64  (2.42,5.48) | 3.64  (2.42,5.47) | 43.94 | 1.86  (1.12) | 3.63  (2.41) |
| Investigations | Blood sodium decreased | 22 | 3.52  (2.32,5.35) | 3.52  (2.32,5.34) | 39.63 | 1.81  (1.06) | 3.52  (2.31) |
| Respiratory, thoracic and mediastinal disorders | Orthopnoea | 22 | 20.54  (13.50,31.25) | 20.52  (13.49,31.20) | 405.60 | 4.35  (2.86) | 20.38  (13.40) |
| Cardiac disorders | Cardiac failure chronic | 21 | 15.04  (9.79,23.09) | 15.02  (9.79,23.06) | 273.42 | 3.90  (2.58) | 14.95  (9.73) |
| Investigations | Creatinine renal clearance decreased | 21 | 16.38  (10.66,25.16) | 16.36  (10.66,25.12) | 301.19 | 4.02  (2.65) | 16.27  (10.60) |
| Renal and urinary disorders | Microalbuminuria | 20 | 79.43  (50.92,123.89) | 79.35  (50.89,123.72) | 1505.32 | 6.27  (3.42) | 77.23  (49.51) |
| Vascular disorders | Circulatory collapse | 19 | 3.34  (2.13,5.23) | 3.33  (2.13,5.23) | 31.02 | 1.74  (0.93) | 3.33  (2.12) |
| Metabolism and nutrition disorders | Gout | 19 | 3.20  (2.04,5.03) | 3.20  (2.04,5.02) | 28.75 | 1.68  (0.88) | 3.20  (2.04) |
| Cardiac disorders | Ventricular extrasystoles | 19 | 5.41  (3.45,8.49) | 5.41  (3.45,8.48) | 68.15 | 2.43  (1.50) | 5.40  (3.44) |
| General disorders and administration site conditions | Sudden cardiac death | 19 | 16.22  (10.33,25.46) | 16.20  (10.32,25.43) | 269.49 | 4.01  (2.55) | 16.12  (10.26) |
| Surgical and medical procedures | Dialysis | 19 | 4.01  (2.56,6.29) | 4.01  (2.56,6.29) | 42.86 | 2.00  (1.15) | 4.00  (2.55) |
| Cardiac disorders | Cardiogenic shock | 18 | 3.91  (2.47,6.22) | 3.91  (2.46,6.21) | 38.97 | 1.97  (1.10) | 3.91  (2.46) |
| Investigations | Brain natriuretic peptide increased | 18 | 17.99  (11.32,28.61) | 17.98  (11.31,28.57) | 286.83 | 4.16  (2.58) | 17.87  (11.24) |
| Vascular disorders | Infarction | 18 | 7.76  (4.88,12.33) | 7.75  (4.88,12.31) | 105.61 | 2.95  (1.85) | 7.74  (4.87) |
| Metabolism and nutrition disorders | Hypovolaemia | 17 | 8.68  (5.39,13.97) | 8.67  (5.39,13.95) | 115.01 | 3.11  (1.92) | 8.65  (5.37) |
| Cardiac disorders | Tricuspid valve incompetence | 17 | 6.54  (4.06,10.53) | 6.53  (4.06,10.51) | 79.51 | 2.71  (1.64) | 6.52  (4.05) |
| Respiratory, thoracic and mediastinal disorders | Acute pulmonary oedema | 16 | 8.55  (5.23,13.97) | 8.55  (5.23,13.96) | 106.30 | 3.09  (1.86) | 8.52  (5.22) |
| Infections and infestations | Gastroenteritis | 16 | 3.32  (2.04,5.43) | 3.32  (2.04,5.42) | 25.96 | 1.73  (0.85) | 3.32  (2.03) |
| Gastrointestinal disorders | Lip oedema | 16 | 11.44  (7.00,18.69) | 11.43  (7.00,18.67) | 151.64 | 3.51  (2.12) | 11.39  (6.97) |
| Respiratory, thoracic and mediastinal disorders | Pulmonary congestion | 16 | 3.62  (2.22,5.91) | 3.62  (2.22,5.91) | 30.29 | 1.85  (0.95) | 3.62  (2.21) |
| Nervous system disorders | Sensory loss | 16 | 6.04  (3.70,9.87) | 6.04  (3.70,9.86) | 67.10 | 2.59  (1.52) | 6.03  (3.69) |
| Cardiac disorders | Angina unstable | 15 | 6.59  (3.97,10.94) | 6.59  (3.97,10.93) | 70.93 | 2.72  (1.56) | 6.57  (3.96) |
| Investigations | Electrocardiogram QRS complex prolonged | 15 | 9.18  (5.53,15.24) | 9.17  (5.53,15.23) | 108.91 | 3.19  (1.88) | 9.15  (5.51) |
| Investigations | Renin increased | 15 | 175.73  (104.30,296.07) | 175.60  (104.27,295.74) | 2452.73 | 7.37  (3.13) | 165.45  (98.20) |
| General disorders and administration site conditions | Sudden death | 15 | 4.17  (2.51,6.92) | 4.16  (2.51,6.91) | 36.03 | 2.06  (1.07) | 4.16  (2.51) |
| Renal and urinary disorders | Nephropathy | 14 | 4.46  (2.64,7.53) | 4.45  (2.64,7.52) | 37.46 | 2.15  (1.11) | 4.45  (2.63) |
| Renal and urinary disorders | Oliguria | 14 | 6.78  (4.01,11.46) | 6.78  (4.01,11.45) | 68.81 | 2.76  (1.54) | 6.77  (4.00) |
| Renal and urinary disorders | Renal cyst | 14 | 5.26  (3.11,8.88) | 5.25  (3.11,8.87) | 48.13 | 2.39  (1.29) | 5.25  (3.10) |
| Gastrointestinal disorders | Tongue oedema | 14 | 10.95  (6.48,18.52) | 10.95  (6.48,18.50) | 126.07 | 3.45  (1.97) | 10.91  (6.45) |
| Cardiac disorders | Ventricular fibrillation | 14 | 3.81  (2.25,6.43) | 3.80  (2.25,6.42) | 28.89 | 1.93  (0.93) | 3.80  (2.25) |
| Cardiac disorders | Left ventricular dysfunction | 14 | 6.44  (3.81,10.88) | 6.44  (3.81,10.87) | 64.14 | 2.68  (1.49) | 6.42  (3.80) |
| Vascular disorders | Arterial occlusive disease | 14 | 5.33  (3.16,9.01) | 5.33  (3.15,9.00) | 49.14 | 2.41  (1.30) | 5.32  (3.15) |
| Renal and urinary disorders | Azotaemia | 13 | 9.94  (5.77,17.15) | 9.94  (5.77,17.13) | 104.15 | 3.31  (1.83) | 9.91  (5.75) |
| Investigations | Blood creatine increased | 13 | 8.88  (5.15,15.31) | 8.87  (5.15,15.29) | 90.54 | 3.15  (1.73) | 8.85  (5.13) |
| Gastrointestinal disorders | Oedema mouth | 13 | 11.46  (6.65,19.77) | 11.46  (6.65,19.75) | 123.58 | 3.51  (1.94) | 11.41  (6.62) |
| Cardiac disorders | Acute coronary syndrome | 13 | 4.60  (2.67,7.93) | 4.60  (2.67,7.92) | 36.57 | 2.20  (1.10) | 4.59  (2.67) |
| General disorders and administration site conditions | Concomitant disease aggravated | 12 | 5.87  (3.33,10.34) | 5.86  (3.33,10.33) | 48.33 | 2.55  (1.29) | 5.85  (3.32) |
| Cardiac disorders | Coronary artery stenosis | 12 | 6.95  (3.94,12.25) | 6.95  (3.94,12.24) | 60.95 | 2.79  (1.45) | 6.93  (3.93) |
| Renal and urinary disorders | Nephrotic syndrome | 12 | 5.35  (3.04,9.42) | 5.35  (3.03,9.42) | 42.32 | 2.42  (1.20) | 5.34  (3.03) |
| Gastrointestinal disorders | Odynophagia | 12 | 6.26  (3.55,11.03) | 6.26  (3.55,11.02) | 52.89 | 2.64  (1.35) | 6.25  (3.54) |
| Psychiatric disorders | Personality disorder | 12 | 14.25  (8.08,25.13) | 14.24  (8.08,25.11) | 147.01 | 3.83  (2.01) | 14.18  (8.04) |
| Respiratory, thoracic and mediastinal disorders | Rales | 12 | 6.12  (3.47,10.78) | 6.12  (3.47,10.78) | 51.26 | 2.61  (1.33) | 6.11  (3.46) |
| Cardiac disorders | Tachyarrhythmia | 12 | 14.36  (8.14,25.33) | 14.36  (8.14,25.31) | 148.36 | 3.84  (2.02) | 14.29  (8.10) |
| Gastrointestinal disorders | Large intestine polyp | 12 | 4.09  (2.32,7.21) | 4.09  (2.32,7.20) | 27.96 | 2.03  (0.92) | 4.08  (2.32) |
| Nervous system disorders | Motor dysfunction | 12 | 3.63  (2.06,6.40) | 3.63  (2.06,6.39) | 22.84 | 1.86  (0.79) | 3.63  (2.06) |
| Cardiac disorders | Atrial flutter | 11 | 4.12  (2.28,7.44) | 4.12  (2.28,7.44) | 25.92 | 2.04  (0.87) | 4.11  (2.28) |
| Cardiac disorders | Atrioventricular block | 11 | 4.28  (2.37,7.73) | 4.27  (2.37,7.72) | 27.56 | 2.09  (0.91) | 4.27  (2.36) |
| Surgical and medical procedures | Cardiac pacemaker insertion | 11 | 5.53  (3.06,9.99) | 5.53  (3.06,9.98) | 40.70 | 2.46  (1.17) | 5.52  (3.05) |
| Nervous system disorders | Carotid artery thrombosis | 11 | 44.22  (24.37,80.23) | 44.20  (24.37,80.16) | 457.33 | 5.44  (2.42) | 43.54  (24.00) |
| Endocrine disorders | Hyperparathyroidism secondary | 11 | 12.93  (7.15,23.38) | 12.92  (7.15,23.36) | 120.43 | 3.69  (1.86) | 12.87  (7.11) |
| Respiratory, thoracic and mediastinal disorders | Stridor | 11 | 11.32  (6.26,20.47) | 11.31  (6.26,20.45) | 103.01 | 3.49  (1.77) | 11.27  (6.23) |
| Cardiac disorders | Dilated cardiomyopathy | 11 | 6.28  (3.47,11.34) | 6.27  (3.47,11.34) | 48.67 | 2.65  (1.29) | 6.26  (3.47) |
| Investigations | Blood pressure systolic decreased | 10 | 6.71  (3.60,12.47) | 6.70  (3.60,12.47) | 48.41 | 2.74  (1.27) | 6.69  (3.60) |
| Cardiac disorders | Bundle branch block left | 10 | 6.95  (3.74,12.93) | 6.95  (3.73,12.92) | 50.77 | 2.79  (1.30) | 6.93  (3.73) |
| Eye disorders | Conjunctival haemorrhage | 10 | 7.82  (4.20,14.54) | 7.81  (4.20,14.53) | 59.25 | 2.96  (1.40) | 7.79  (4.19) |
| Investigations | Electrocardiogram abnormal | 10 | 3.77  (2.03,7.01) | 3.77  (2.03,7.00) | 20.30 | 1.91  (0.72) | 3.76  (2.02) |
| Nervous system disorders | Neurologic neglect syndrome | 10 | 60.68  (32.43,113.55) | 60.66  (32.43,113.46) | 574.48 | 5.89  (2.35) | 59.41  (31.75) |
| Cardiac disorders | Diastolic dysfunction | 10 | 9.86  (5.30,18.36) | 9.86  (5.30,18.34) | 79.35 | 3.30  (1.57) | 9.83  (5.28) |
| Psychiatric disorders | Adjustment disorder | 10 | 24.92  (13.37,46.45) | 24.91  (13.37,46.42) | 227.52 | 4.63  (2.09) | 24.70  (13.25) |
| Investigations | Blood pressure diastolic increased | 9 | 5.92  (3.08,11.39) | 5.92  (3.08,11.38) | 36.72 | 2.56  (1.07) | 5.91  (3.07) |
| Metabolism and nutrition disorders | Hypertriglyceridaemia | 9 | 4.68  (2.43,9.01) | 4.68  (2.43,9.00) | 26.02 | 2.23  (0.86) | 4.68  (2.43) |
| Cardiac disorders | Supraventricular extrasystoles | 9 | 7.81  (4.06,15.03) | 7.81  (4.06,15.02) | 53.28 | 2.96  (1.30) | 7.79  (4.05) |
| Cardiac disorders | Bradyarrhythmia | 9 | 16.73  (8.69,32.22) | 16.72  (8.69,32.20) | 132.27 | 4.06  (1.78) | 16.63  (8.64) |
| Investigations | Protein urine present | 9 | 5.60  (2.91,10.77) | 5.60  (2.91,10.76) | 33.92 | 2.48  (1.02) | 5.59  (2.91) |
| Blood and lymphatic system disorders | Nephrogenic anaemia | 9 | 5.64  (2.93,10.85) | 5.64  (2.93,10.85) | 34.30 | 2.49  (1.03) | 5.63  (2.93) |
| Cardiac disorders | Atrioventricular block first degree | 8 | 5.39  (2.70,10.79) | 5.39  (2.69,10.79) | 28.56 | 2.43  (0.89) | 5.38  (2.69) |
| Nervous system disorders | Diabetic neuropathy | 8 | 4.83  (2.41,9.66) | 4.83  (2.41,9.66) | 24.24 | 2.27  (0.80) | 4.82  (2.41) |
| Eye disorders | Diabetic retinopathy | 8 | 7.15  (3.57,14.32) | 7.15  (3.57,14.31) | 42.21 | 2.83  (1.12) | 7.13  (3.56) |
| Metabolism and nutrition disorders | Hyperuricaemia | 8 | 5.93  (2.96,11.87) | 5.93  (2.96,11.86) | 32.72 | 2.57  (0.97) | 5.92  (2.96) |
| Respiratory, thoracic and mediastinal disorders | Nocturnal dyspnoea | 8 | 19.21  (9.58,38.51) | 19.21  (9.58,38.49) | 137.13 | 4.25  (1.70) | 19.08  (9.52) |
| General disorders and administration site conditions | Non-cardiac chest pain | 8 | 7.31  (3.65,14.63) | 7.31  (3.65,14.62) | 43.45 | 2.87  (1.14) | 7.29  (3.64) |
| Surgical and medical procedures | Endotracheal intubation | 8 | 7.93  (3.96,15.87) | 7.93  (3.96,15.86) | 48.29 | 2.98  (1.20) | 7.91  (3.95) |
| Surgical and medical procedures | Vascular graft | 8 | 7.81  (3.90,15.63) | 7.80  (3.90,15.62) | 47.33 | 2.96  (1.19) | 7.79  (3.89) |
| Vascular disorders | Aortic stenosis | 7 | 6.03  (2.87,12.65) | 6.02  (2.87,12.64) | 29.27 | 2.59  (0.86) | 6.01  (2.86) |
| Cardiac disorders | Aortic valve incompetence | 7 | 4.91  (2.34,10.32) | 4.91  (2.34,10.31) | 21.78 | 2.29  (0.70) | 4.91  (2.34) |
| Investigations | Blood sodium increased | 7 | 7.71  (3.67,16.19) | 7.71  (3.67,16.19) | 40.76 | 2.94  (1.04) | 7.69  (3.66) |
| Nervous system disorders | Carotid artery stenosis | 7 | 5.85  (2.79,12.29) | 5.85  (2.79,12.28) | 28.09 | 2.55  (0.84) | 5.84  (2.78) |
| Respiratory, thoracic and mediastinal disorders | Dyspnoea paroxysmal nocturnal | 7 | 27.74  (13.18,58.41) | 27.73  (13.18,58.38) | 178.65 | 4.78  (1.65) | 27.48  (13.05) |
| Investigations | Protein total decreased | 7 | 5.54  (2.64,11.63) | 5.54  (2.64,11.62) | 25.98 | 2.47  (0.80) | 5.53  (2.63) |
| Renal and urinary disorders | Renal atrophy | 7 | 16.50  (7.85,34.70) | 16.50  (7.85,34.68) | 101.33 | 4.04  (1.46) | 16.41  (7.80) |
| Surgical and medical procedures | Resuscitation | 7 | 12.44  (5.92,26.14) | 12.44  (5.92,26.13) | 73.31 | 3.63  (1.33) | 12.39  (5.90) |
| Investigations | Renal function test abnormal | 7 | 4.66  (2.22,9.79) | 4.66  (2.22,9.78) | 20.11 | 2.22  (0.65) | 4.66  (2.22) |
| Neoplasms benign, malignant and unspecified (incl cysts and polyps) | Colorectal adenoma | 7 | 15.24  (7.25,32.04) | 15.24  (7.25,32.02) | 92.62 | 3.92  (1.43) | 15.16  (7.21) |
| Investigations | Albumin urine present | 6 | 48.64  (21.70,109.03) | 48.63  (21.70,108.97) | 275.19 | 5.58  (1.54) | 47.83  (21.34) |
| Cardiac disorders | Atrioventricular block second degree | 6 | 5.87  (2.63,13.07) | 5.87  (2.63,13.07) | 24.17 | 2.55  (0.70) | 5.86  (2.63) |
| Respiratory, thoracic and mediastinal disorders | Dyspnoea at rest | 6 | 6.43  (2.89,14.33) | 6.43  (2.89,14.33) | 27.46 | 2.68  (0.76) | 6.42  (2.88) |
| Cardiac disorders | Left ventricular failure | 6 | 5.24  (2.35,11.67) | 5.24  (2.35,11.66) | 20.52 | 2.39  (0.61) | 5.23  (2.35) |
| Cardiac disorders | Ischaemic cardiomyopathy | 6 | 5.57  (2.50,12.41) | 5.57  (2.50,12.40) | 22.44 | 2.47  (0.66) | 5.56  (2.49) |
| Cardiac disorders | Ventricular hypokinesia | 6 | 5.53  (2.48,12.31) | 5.52  (2.48,12.30) | 22.19 | 2.46  (0.65) | 5.52  (2.48) |
| Investigations | Creatinine urine increased | 6 | 63.24  (28.16,142.02) | 63.22  (28.15,141.95) | 359.41 | 5.95  (1.57) | 61.86  (27.54) |
| Cardiac disorders | Left atrial dilatation | 6 | 13.89  (6.23,30.99) | 13.89  (6.23,30.97) | 71.41 | 3.79  (1.19) | 13.83  (6.20) |
| Investigations | Blood urea decreased | 5 | 11.91  (4.95,28.66) | 11.90  (4.95,28.65) | 49.73 | 3.57  (0.90) | 11.86  (4.93) |
| Congenital, familial and genetic disorders | Conjoined twins | 5 | 159.86  (64.93,393.55) | 159.82  (64.93,393.37) | 747.15 | 7.24  (1.32) | 151.37  (61.49) |
| General disorders and administration site conditions | Crepitations | 5 | 5.00  (2.08,12.02) | 5.00  (2.08,12.01) | 15.95 | 2.32  (0.40) | 4.99  (2.07) |
| Respiratory, thoracic and mediastinal disorders | Laryngospasm | 5 | 5.52  (2.29,13.27) | 5.52  (2.29,13.27) | 18.46 | 2.46  (0.47) | 5.51  (2.29) |
| Vascular disorders | Malignant hypertension | 5 | 17.16  (7.12,41.35) | 17.16  (7.12,41.33) | 75.63 | 4.09  (1.03) | 17.06  (7.08) |
| Renal and urinary disorders | Nephrosclerosis | 5 | 12.83  (5.33,30.89) | 12.83  (5.33,30.87) | 54.28 | 3.67  (0.93) | 12.77  (5.31) |
| Neoplasms benign, malignant and unspecified (incl cysts and polyps) | Rectal cancer | 5 | 4.86  (2.02,11.68) | 4.85  (2.02,11.67) | 15.28 | 2.28  (0.38) | 4.85  (2.02) |
| General disorders and administration site conditions | Sense of oppression | 5 | 12.65  (5.25,30.45) | 12.64  (5.25,30.43) | 53.38 | 3.65  (0.92) | 12.59  (5.23) |
| Respiratory, thoracic and mediastinal disorders | Suffocation feeling | 5 | 6.06  (2.52,14.57) | 6.06  (2.52,14.56) | 21.06 | 2.60  (0.54) | 6.04  (2.51) |
| Neoplasms benign, malignant and unspecified (incl cysts and polyps) | Thyroid neoplasm | 5 | 5.29  (2.20,12.72) | 5.29  (2.20,12.72) | 17.36 | 2.40  (0.44) | 5.28  (2.20) |
| Neoplasms benign, malignant and unspecified (incl cysts and polyps) | Transitional cell carcinoma | 5 | 11.72  (4.87,28.21) | 11.72  (4.87,28.20) | 48.81 | 3.55  (0.89) | 11.67  (4.85) |
| Cardiac disorders | Ventricular hypertrophy | 5 | 5.62  (2.34,13.52) | 5.62  (2.34,13.51) | 18.95 | 2.49  (0.49) | 5.61  (2.33) |
| Surgical and medical procedures | Implantable defibrillator insertion | 5 | 11.26  (4.68,27.11) | 11.26  (4.68,27.10) | 46.57 | 3.49  (0.87) | 11.22  (4.66) |
| Neoplasms benign, malignant and unspecified (incl cysts and polyps) | Lung cancer metastatic | 5 | 5.11  (2.13,12.30) | 5.11  (2.13,12.29) | 16.51 | 2.35  (0.42) | 5.11  (2.12) |
| Surgical and medical procedures | Coronary angioplasty | 5 | 33.32  (13.80,80.47) | 33.31  (13.80,80.43) | 154.89 | 5.04  (1.19) | 32.94  (13.64) |
| Investigations | Electrocardiogram T wave abnormal | 5 | 9.64  (4.01,23.19) | 9.64  (4.01,23.18) | 38.57 | 3.26  (0.80) | 9.61  (3.99) |
| Nervous system disorders | Tongue biting | 5 | 7.25  (3.02,17.45) | 7.25  (3.02,17.44) | 26.89 | 2.86  (0.65) | 7.24  (3.01) |
| Nervous system disorders | Vascular dementia | 5 | 17.54  (7.28,42.27) | 17.54  (7.28,42.25) | 77.50 | 4.12  (1.04) | 17.44  (7.24) |
| Vascular disorders | Aortic disorder | 5 | 18.36  (7.62,44.23) | 18.35  (7.62,44.21) | 81.51 | 4.19  (1.05) | 18.24  (7.57) |
| Respiratory, thoracic and mediastinal disorders | Pulmonary vascular disorder | 5 | 30.53  (12.65,73.70) | 30.52  (12.65,73.67) | 141.26 | 4.92  (1.18) | 30.21  (12.51) |
| Surgical and medical procedures | Percutaneous coronary intervention | 5 | 52.31  (21.59,126.69) | 52.29  (21.59,126.64) | 247.02 | 5.68  (1.26) | 51.37  (21.21) |
| Investigations | Blood albumin increased | 4 | 13.42  (5.02,35.85) | 13.42  (5.03,35.83) | 45.76 | 3.74  (0.65) | 13.36  (5.00) |
| Investigations | Blood aldosterone increased | 4 | 70.69  (26.21,190.66) | 70.68  (26.21,190.59) | 268.11 | 6.11  (0.93) | 68.99  (25.58) |
| Cardiac disorders | Cardiac hypertrophy | 4 | 11.07  (4.15,29.56) | 11.07  (4.15,29.55) | 36.50 | 3.46  (0.58) | 11.03  (4.13) |
| Cardiac disorders | Dilatation ventricular | 4 | 5.40  (2.03,14.41) | 5.40  (2.03,14.40) | 14.32 | 2.43  (0.23) | 5.39  (2.02) |
| Gastrointestinal disorders | Enterocolitis haemorrhagic | 4 | 7.95  (2.98,21.21) | 7.95  (2.98,21.20) | 24.22 | 2.99  (0.44) | 7.93  (2.97) |
| Metabolism and nutrition disorders | Hyperphosphataemia | 4 | 5.95  (2.23,15.88) | 5.95  (2.23,15.87) | 16.44 | 2.57  (0.29) | 5.94  (2.23) |
| Endocrine disorders | Primary hyperaldosteronism | 4 | 77.95  (28.87,210.51) | 77.94  (28.87,210.43) | 295.70 | 6.25  (0.94) | 75.89  (28.10) |
| Congenital, familial and genetic disorders | Truncus arteriosus persistent | 4 | 66.56  (24.69,179.39) | 66.54  (24.69,179.32) | 252.33 | 6.02  (0.93) | 65.05  (24.13) |
| Renal and urinary disorders | Nephroangiosclerosis | 4 | 74.88  (27.74,202.10) | 74.86  (27.74,202.02) | 284.03 | 6.19  (0.93) | 72.97  (27.03) |
| Surgical and medical procedures | Catheter placement | 4 | 6.48  (2.43,17.29) | 6.48  (2.43,17.28) | 18.50 | 2.69  (0.33) | 6.47  (2.42) |
| Congenital, familial and genetic disorders | Congenital bladder anomaly | 4 | 86.88  (32.12,234.97) | 86.86  (32.12,234.88) | 329.44 | 6.40  (0.94) | 84.32  (31.18) |
| Renal and urinary disorders | Renal mass | 4 | 5.86  (2.20,15.64) | 5.86  (2.20,15.63) | 16.10 | 2.55  (0.28) | 5.85  (2.19) |
| Injury, poisoning and procedural complications | Post procedural haematoma | 4 | 6.93  (2.60,18.48) | 6.93  (2.60,18.47) | 20.23 | 2.79  (0.37) | 6.91  (2.59) |
| Congenital, familial and genetic disorders | Intestinal malrotation | 4 | 44.98  (16.75,120.80) | 44.98  (16.75,120.75) | 169.32 | 5.47  (0.89) | 44.29  (16.49) |
| Gastrointestinal disorders | Lip exfoliation | 4 | 9.16  (3.43,24.46) | 9.16  (3.43,24.45) | 28.99 | 3.19  (0.50) | 9.14  (3.42) |
| Congenital, familial and genetic disorders | Urachal abnormality | 4 | 284.53  (101.79,795.34) | 284.48  (101.79,795.04) | 1027.20 | 8.02  (0.94) | 258.71  (92.55) |
| Injury, poisoning and procedural complications | Heat illness | 4 | 17.76  (6.64,47.46) | 17.75  (6.64,47.44) | 62.84 | 4.14  (0.73) | 17.65  (6.60) |
| Congenital, familial and genetic disorders | Persistent cloaca | 4 | 344.89  (122.17,973.60) | 344.82  (122.17,973.23) | 1223.04 | 8.27  (0.93) | 307.65  (108.98) |
| Investigations | Angiocardiogram | 4 | 77.42  (28.67,209.06) | 77.41  (28.67,208.98) | 293.69 | 6.24  (0.94) | 75.38  (27.92) |
| Hepatobiliary disorders | Congestive hepatopathy | 4 | 8.39  (3.15,22.40) | 8.39  (3.15,22.39) | 25.97 | 3.07  (0.47) | 8.37  (3.14) |
| Renal and urinary disorders | Albuminuria | 3 | 17.67  (5.68,54.99) | 17.67  (5.68,54.97) | 46.89 | 4.13  (0.32) | 17.57  (5.65) |
| Cardiac disorders | Aortic valve sclerosis | 3 | 11.71  (3.77,36.39) | 11.71  (3.77,36.38) | 29.26 | 3.54  (0.22) | 11.66  (3.75) |
| Musculoskeletal and connective tissue disorders | Gouty arthritis | 3 | 11.11  (3.58,34.54) | 11.11  (3.58,34.53) | 27.50 | 3.47  (0.21) | 11.07  (3.56) |
| General disorders and administration site conditions | Gravitational oedema | 3 | 12.55  (4.04,39.02) | 12.55  (4.04,39.01) | 31.75 | 3.64  (0.24) | 12.50  (4.02) |
| Infections and infestations | Orchitis | 3 | 8.69  (2.80,27.00) | 8.69  (2.80,26.99) | 20.36 | 3.12  (0.13) | 8.67  (2.79) |
| Vascular disorders | Paradoxical pressor response | 3 | 775.96  (216.46,2781.64) | 775.84  (216.46,2780.82) | 1824.06 | 9.25  (0.37) | 609.81  (170.11) |
| Nervous system disorders | Tongue paralysis | 3 | 12.82  (4.12,39.84) | 12.81  (4.12,39.83) | 32.53 | 3.67  (0.25) | 12.76  (4.10) |
| Infections and infestations | Tracheobronchitis | 3 | 11.41  (3.67,35.46) | 11.41  (3.67,35.45) | 28.38 | 3.51  (0.22) | 11.37  (3.66) |
| Investigations | Venous pressure jugular increased | 3 | 24.39  (7.83,75.99) | 24.38  (7.83,75.97) | 66.70 | 4.60  (0.38) | 24.18  (7.76) |
| Infections and infestations | Vestibular neuronitis | 3 | 16.94  (5.44,52.69) | 16.93  (5.44,52.68) | 44.71 | 4.07  (0.31) | 16.84  (5.41) |
| Gastrointestinal disorders | Tongue haemorrhage | 3 | 7.71  (2.48,23.95) | 7.71  (2.48,23.94) | 17.47 | 2.94  (0.08) | 7.69  (2.48) |
| General disorders and administration site conditions | Therapy responder | 3 | 18.32  (5.89,57.00) | 18.31  (5.89,56.99) | 48.79 | 4.19  (0.33) | 18.20  (5.85) |
| Reproductive system and breast disorders | Organic erectile dysfunction | 3 | 20.82  (6.69,64.82) | 20.82  (6.69,64.80) | 56.18 | 4.37  (0.35) | 20.67  (6.64) |
| Investigations | Electrocardiogram repolarisation abnormality | 3 | 11.38  (3.66,35.37) | 11.38  (3.66,35.36) | 28.29 | 3.50  (0.21) | 11.34  (3.65) |
| Injury, poisoning and procedural complications | Coronary artery restenosis | 3 | 14.27  (4.59,44.39) | 14.27  (4.59,44.37) | 36.84 | 3.83  (0.28) | 14.21  (4.57) |
| Investigations | Lymphocyte stimulation test positive | 3 | 10.42  (3.35,32.38) | 10.42  (3.35,32.37) | 25.46 | 3.38  (0.19) | 10.39  (3.34) |
| Nervous system disorders | Ischaemic cerebral infarction | 3 | 7.90  (2.54,24.52) | 7.89  (2.54,24.52) | 18.01 | 2.98  (0.09) | 7.88  (2.54) |
| Congenital, familial and genetic disorders | Congenital cardiovascular anomaly | 3 | 10.70  (3.44,33.24) | 10.69  (3.44,33.23) | 26.27 | 3.41  (0.20) | 10.66  (3.43) |
| Renal and urinary disorders | Glomerulosclerosis | 3 | 14.11  (4.54,43.87) | 14.11  (4.54,43.86) | 36.35 | 3.81  (0.27) | 14.04  (4.52) |
| General disorders and administration site conditions | Effusion | 3 | 6.54  (2.11,20.31) | 6.54  (2.11,20.30) | 14.05 | 2.71  (0.01) | 6.53  (2.10) |
| Congenital, familial and genetic disorders | Cardiac septal defect | 3 | 12.65  (4.07,39.31) | 12.64  (4.07,39.30) | 32.03 | 3.65  (0.24) | 12.59  (4.05) |
| Congenital, familial and genetic disorders | Congenital musculoskeletal disorder of limbs | 3 | 9.89  (3.18,30.73) | 9.89  (3.18,30.72) | 23.89 | 3.30  (0.17) | 9.86  (3.17) |

Note1:ranked by Reports

Note2:Signals are detected when all the following criteria are met:a ≥ 3, PRR ≥2 and Chi-Square ≥ 4, lower limit of 95% CI of ROR > 1, IC025 > 0, EBGM05 > 2.

TABLE 4 Signal strength of adverse events at the Preferred Term(PT) level ranked by ROR

| **System Organ Class(SOC)** | **Preferred Term(PT)** | **Case reports** | **ROR (95% CI)** | **PRR (95% CI)** | **Chi Square** | **IC (IC025)** | **EBGM (EBGM05)** |
| --- | --- | --- | --- | --- | --- | --- | --- |
| Vascular disorders | Paradoxical pressor response | 3 | 775.96  (216.46,2781.64) | 775.84  (216.46,2780.82) | 1824.06 | 9.25  (0.37) | 609.81  (170.11) |
| Investigations | Urine albumin/creatinine ratio increased | 58 | 433.15  (328.50,571.14) | 431.93  (327.79,569.14) | 21649.0 | 8.55  (5.27) | 375.12  (284.49) |
| Congenital, familial and genetic disorders | Persistent cloaca | 4 | 344.89  (122.17,973.60) | 344.82  (122.17,973.23) | 1223.04 | 8.27  (0.93) | 307.65  (108.98) |
| Congenital, familial and genetic disorders | Urachal abnormality | 4 | 284.53  (101.79,795.34) | 284.48  (101.79,795.04) | 1027.20 | 8.02  (0.94) | 258.71  (92.55) |
| Investigations | Renin increased | 15 | 175.73  (104.30,296.07) | 175.60  (104.27,295.74) | 2452.73 | 7.37  (3.13) | 165.45  (98.20) |
| Congenital, familial and genetic disorders | Conjoined twins | 5 | 159.86  (64.93,393.55) | 159.82  (64.93,393.37) | 747.15 | 7.24  (1.32) | 151.37  (61.49) |
| General disorders and administration site conditions | Concomitant disease progression | 89 | 139.26  (112.52,172.35) | 138.65  (112.13,171.45) | 11598.0 | 7.05  (5.44) | 132.26  (106.86) |
| Vascular disorders | Blood pressure inadequately controlled | 185 | 93.62  (80.82,108.46) | 92.79  (80.20,107.35) | 16268.4 | 6.49  (5.71) | 89.89  (77.59) |
| Congenital, familial and genetic disorders | Congenital bladder anomaly | 4 | 86.88  (32.12,234.97) | 86.86  (32.12,234.88) | 329.44 | 6.40  (0.94) | 84.32  (31.18) |
| Renal and urinary disorders | Microalbuminuria | 20 | 79.43  (50.92,123.89) | 79.35  (50.89,123.72) | 1505.32 | 6.27  (3.42) | 77.23  (49.51) |
| Endocrine disorders | Primary hyperaldosteronism | 4 | 77.95  (28.87,210.51) | 77.94  (28.87,210.43) | 295.70 | 6.25  (0.94) | 75.89  (28.10) |
| Investigations | Angiocardiogram | 4 | 77.42  (28.67,209.06) | 77.41  (28.67,208.98) | 293.69 | 6.24  (0.94) | 75.38  (27.92) |
| Renal and urinary disorders | Nephroangiosclerosis | 4 | 74.88  (27.74,202.10) | 74.86  (27.74,202.02) | 284.03 | 6.19  (0.93) | 72.97  (27.03) |
| Investigations | Blood aldosterone increased | 4 | 70.69  (26.21,190.66) | 70.68  (26.21,190.59) | 268.11 | 6.11  (0.93) | 68.99  (25.58) |
| Congenital, familial and genetic disorders | Truncus arteriosus persistent | 4 | 66.56  (24.69,179.39) | 66.54  (24.69,179.32) | 252.33 | 6.02  (0.93) | 65.05  (24.13) |
| Investigations | Creatinine urine increased | 6 | 63.24  (28.16,142.02) | 63.22  (28.15,141.95) | 359.41 | 5.95  (1.57) | 61.86  (27.54) |
| Nervous system disorders | Neurologic neglect syndrome | 10 | 60.68  (32.43,113.55) | 60.66  (32.43,113.46) | 574.48 | 5.89  (2.35) | 59.41  (31.75) |
| Surgical and medical procedures | Percutaneous coronary intervention | 5 | 52.31  (21.59,126.69) | 52.29  (21.59,126.64) | 247.02 | 5.68  (1.26) | 51.37  (21.21) |
| Renal and urinary disorders | Renal artery stenosis | 23 | 49.32  (32.65,74.50) | 49.27  (32.63,74.39) | 1069.16 | 5.60  (3.43) | 48.45  (32.07) |
| Investigations | Albumin urine present | 6 | 48.64  (21.70,109.03) | 48.63  (21.70,108.97) | 275.19 | 5.58  (1.54) | 47.83  (21.34) |
| Congenital, familial and genetic disorders | Intestinal malrotation | 4 | 44.98  (16.75,120.80) | 44.98  (16.75,120.75) | 169.32 | 5.47  (0.89) | 44.29  (16.49) |
| Nervous system disorders | Carotid artery thrombosis | 11 | 44.22  (24.37,80.23) | 44.20  (24.37,80.16) | 457.33 | 5.44  (2.42) | 43.54  (24.00) |
| Renal and urinary disorders | Diabetic nephropathy | 30 | 40.60  (28.31,58.23) | 40.54  (28.28,58.12) | 1140.81 | 5.32  (3.62) | 39.99  (27.88) |
| Surgical and medical procedures | Coronary angioplasty | 5 | 33.32  (13.80,80.47) | 33.31  (13.80,80.43) | 154.89 | 5.04  (1.19) | 32.94  (13.64) |
| Respiratory, thoracic and mediastinal disorders | Pulmonary vascular disorder | 5 | 30.53  (12.65,73.70) | 30.52  (12.65,73.67) | 141.26 | 4.92  (1.18) | 30.21  (12.51) |
| Respiratory, thoracic and mediastinal disorders | Dyspnoea paroxysmal nocturnal | 7 | 27.74  (13.18,58.41) | 27.73  (13.18,58.38) | 178.65 | 4.78  (1.65) | 27.48  (13.05) |
| Psychiatric disorders | Adjustment disorder | 10 | 24.92  (13.37,46.45) | 24.91  (13.37,46.42) | 227.52 | 4.63  (2.09) | 24.70  (13.25) |
| Investigations | Venous pressure jugular increased | 3 | 24.39  (7.83,75.99) | 24.38  (7.83,75.97) | 66.70 | 4.60  (0.38) | 24.18  (7.76) |
| Cardiac disorders | Left ventricular hypertrophy | 32 | 22.53  (15.91,31.92) | 22.50  (15.89,31.85) | 652.29 | 4.48  (3.26) | 22.33  (15.77) |
| Reproductive system and breast disorders | Organic erectile dysfunction | 3 | 20.82  (6.69,64.82) | 20.82  (6.69,64.80) | 56.18 | 4.37  (0.35) | 20.67  (6.64) |
| Respiratory, thoracic and mediastinal disorders | Orthopnoea | 22 | 20.54  (13.50,31.25) | 20.52  (13.49,31.20) | 405.60 | 4.35  (2.86) | 20.38  (13.40) |
| Investigations | Blood pressure diastolic decreased | 48 | 19.65  (14.79,26.11) | 19.60  (14.76,26.03) | 841.79 | 4.28  (3.41) | 19.48  (14.66) |
| Investigations | Blood urea increased | 111 | 19.56  (16.22,23.59) | 19.46  (16.16,23.44) | 1931.30 | 4.27  (3.78) | 19.34  (16.04) |
| Respiratory, thoracic and mediastinal disorders | Nocturnal dyspnoea | 8 | 19.21  (9.58,38.51) | 19.21  (9.58,38.49) | 137.13 | 4.25  (1.70) | 19.08  (9.52) |
| Vascular disorders | Aortic disorder | 5 | 18.36  (7.62,44.23) | 18.35  (7.62,44.21) | 81.51 | 4.19  (1.05) | 18.24  (7.57) |
| General disorders and administration site conditions | Therapy responder | 3 | 18.32  (5.89,57.00) | 18.31  (5.89,56.99) | 48.79 | 4.19  (0.33) | 18.20  (5.85) |
| Investigations | Brain natriuretic peptide increased | 18 | 17.99  (11.32,28.61) | 17.98  (11.31,28.57) | 286.83 | 4.16  (2.58) | 17.87  (11.24) |
| Injury, poisoning and procedural complications | Heat illness | 4 | 17.76  (6.64,47.46) | 17.75  (6.64,47.44) | 62.84 | 4.14  (0.73) | 17.65  (6.60) |
| Renal and urinary disorders | Albuminuria | 3 | 17.67  (5.68,54.99) | 17.67  (5.68,54.97) | 46.89 | 4.13  (0.32) | 17.57  (5.65) |
| Nervous system disorders | Vascular dementia | 5 | 17.54  (7.28,42.27) | 17.54  (7.28,42.25) | 77.50 | 4.12  (1.04) | 17.44  (7.24) |
| Vascular disorders | Malignant hypertension | 5 | 17.16  (7.12,41.35) | 17.16  (7.12,41.33) | 75.63 | 4.09  (1.03) | 17.06  (7.08) |
| Infections and infestations | Vestibular neuronitis | 3 | 16.94  (5.44,52.69) | 16.93  (5.44,52.68) | 44.71 | 4.07  (0.31) | 16.84  (5.41) |
| Cardiac disorders | Bradyarrhythmia | 9 | 16.73  (8.69,32.22) | 16.72  (8.69,32.20) | 132.27 | 4.06  (1.78) | 16.63  (8.64) |
| Vascular disorders | Hypertensive crisis | 61 | 16.62  (12.92,21.39) | 16.57  (12.89,21.31) | 887.69 | 4.04  (3.35) | 16.48  (12.81) |
| Renal and urinary disorders | Renal atrophy | 7 | 16.50  (7.85,34.70) | 16.50  (7.85,34.68) | 101.33 | 4.04  (1.46) | 16.41  (7.80) |
| Investigations | Creatinine renal clearance decreased | 21 | 16.38  (10.66,25.16) | 16.36  (10.66,25.12) | 301.19 | 4.02  (2.65) | 16.27  (10.60) |
| General disorders and administration site conditions | Sudden cardiac death | 19 | 16.22  (10.33,25.46) | 16.20  (10.32,25.43) | 269.49 | 4.01  (2.55) | 16.12  (10.26) |
| Investigations | Blood potassium increased | 86 | 16.14  (13.05,19.96) | 16.08  (13.01,19.86) | 1209.49 | 4.00  (3.46) | 15.99  (12.93) |
| Investigations | Blood creatinine increased | 335 | 15.69  (14.08,17.49) | 15.45  (13.89,17.19) | 4508.81 | 3.94  (3.72) | 15.38  (13.80) |
| Metabolism and nutrition disorders | Hyperkalaemia | 177 | 15.61  (13.45,18.10) | 15.48  (13.36,17.93) | 2385.53 | 3.94  (3.61) | 15.40  (13.28) |
| Eye disorders | Periorbital oedema | 26 | 15.31  (10.41,22.52) | 15.29  (10.41,22.48) | 345.53 | 3.93  (2.76) | 15.22  (10.35) |
| Neoplasms benign, malignant and unspecified (incl cysts and polyps) | Colorectal adenoma | 7 | 15.24  (7.25,32.04) | 15.24  (7.25,32.02) | 92.62 | 3.92  (1.43) | 15.16  (7.21) |
| Investigations | Glomerular filtration rate decreased | 58 | 15.14  (11.69,19.61) | 15.10  (11.67,19.54) | 759.90 | 3.91  (3.22) | 15.03  (11.61) |
| Cardiac disorders | Cardiac failure chronic | 21 | 15.04  (9.79,23.09) | 15.02  (9.79,23.06) | 273.42 | 3.90  (2.58) | 14.95  (9.73) |
| Cardiac disorders | Tachyarrhythmia | 12 | 14.36  (8.14,25.33) | 14.36  (8.14,25.31) | 148.36 | 3.84  (2.02) | 14.29  (8.10) |
| Injury, poisoning and procedural complications | Coronary artery restenosis | 3 | 14.27  (4.59,44.39) | 14.27  (4.59,44.37) | 36.84 | 3.83  (0.28) | 14.21  (4.57) |
| Psychiatric disorders | Personality disorder | 12 | 14.25  (8.08,25.13) | 14.24  (8.08,25.11) | 147.01 | 3.83  (2.01) | 14.18  (8.04) |
| Renal and urinary disorders | Glomerulosclerosis | 3 | 14.11  (4.54,43.87) | 14.11  (4.54,43.86) | 36.35 | 3.81  (0.27) | 14.04  (4.52) |
| Cardiac disorders | Left atrial dilatation | 6 | 13.89  (6.23,30.99) | 13.89  (6.23,30.97) | 71.41 | 3.79  (1.19) | 13.83  (6.20) |
| Investigations | Blood albumin increased | 4 | 13.42  (5.02,35.85) | 13.42  (5.03,35.83) | 45.76 | 3.74  (0.65) | 13.36  (5.00) |
| Endocrine disorders | Hyperparathyroidism secondary | 11 | 12.93  (7.15,23.38) | 12.92  (7.15,23.36) | 120.43 | 3.69  (1.86) | 12.87  (7.11) |
| Renal and urinary disorders | Nephrosclerosis | 5 | 12.83  (5.33,30.89) | 12.83  (5.33,30.87) | 54.28 | 3.67  (0.93) | 12.77  (5.31) |
| Nervous system disorders | Tongue paralysis | 3 | 12.82  (4.12,39.84) | 12.81  (4.12,39.83) | 32.53 | 3.67  (0.25) | 12.76  (4.10) |
| General disorders and administration site conditions | Sense of oppression | 5 | 12.65  (5.25,30.45) | 12.64  (5.25,30.43) | 53.38 | 3.65  (0.92) | 12.59  (5.23) |
| Congenital, familial and genetic disorders | Cardiac septal defect | 3 | 12.65  (4.07,39.31) | 12.64  (4.07,39.30) | 32.03 | 3.65  (0.24) | 12.59  (4.05) |
| General disorders and administration site conditions | Gravitational oedema | 3 | 12.55  (4.04,39.02) | 12.55  (4.04,39.01) | 31.75 | 3.64  (0.24) | 12.50  (4.02) |
| Surgical and medical procedures | Resuscitation | 7 | 12.44  (5.92,26.14) | 12.44  (5.92,26.13) | 73.31 | 3.63  (1.33) | 12.39  (5.90) |
| Investigations | Blood uric acid increased | 23 | 12.23  (8.12,18.43) | 12.22  (8.12,18.40) | 235.97 | 3.61  (2.46) | 12.17  (8.08) |
| Investigations | Blood urea decreased | 5 | 11.91  (4.95,28.66) | 11.90  (4.95,28.65) | 49.73 | 3.57  (0.90) | 11.86  (4.93) |
| Neoplasms benign, malignant and unspecified (incl cysts and polyps) | Transitional cell carcinoma | 5 | 11.72  (4.87,28.21) | 11.72  (4.87,28.20) | 48.81 | 3.55  (0.89) | 11.67  (4.85) |
| Cardiac disorders | Aortic valve sclerosis | 3 | 11.71  (3.77,36.39) | 11.71  (3.77,36.38) | 29.26 | 3.54  (0.22) | 11.66  (3.75) |
| Cardiac disorders | Extrasystoles | 27 | 11.51  (7.88,16.79) | 11.49  (7.88,16.77) | 257.61 | 3.52  (2.51) | 11.45  (7.84) |
| Gastrointestinal disorders | Oedema mouth | 13 | 11.46  (6.65,19.77) | 11.46  (6.65,19.75) | 123.58 | 3.51  (1.94) | 11.41  (6.62) |
| Gastrointestinal disorders | Lip oedema | 16 | 11.44  (7.00,18.69) | 11.43  (7.00,18.67) | 151.64 | 3.51  (2.12) | 11.39  (6.97) |
| Infections and infestations | Tracheobronchitis | 3 | 11.41  (3.67,35.46) | 11.41  (3.67,35.45) | 28.38 | 3.51  (0.22) | 11.37  (3.66) |
| Investigations | Blood pressure systolic increased | 73 | 11.39  (9.05,14.34) | 11.35  (9.02,14.28) | 686.54 | 3.50  (2.97) | 11.31  (8.98) |
| Investigations | Electrocardiogram repolarisation abnormality | 3 | 11.38  (3.66,35.37) | 11.38  (3.66,35.36) | 28.29 | 3.50  (0.21) | 11.34  (3.65) |
| Respiratory, thoracic and mediastinal disorders | Stridor | 11 | 11.32  (6.26,20.47) | 11.31  (6.26,20.45) | 103.01 | 3.49  (1.77) | 11.27  (6.23) |
| Surgical and medical procedures | Implantable defibrillator insertion | 5 | 11.26  (4.68,27.11) | 11.26  (4.68,27.10) | 46.57 | 3.49  (0.87) | 11.22  (4.66) |
| Musculoskeletal and connective tissue disorders | Gouty arthritis | 3 | 11.11  (3.58,34.54) | 11.11  (3.58,34.53) | 27.50 | 3.47  (0.21) | 11.07  (3.56) |
| Cardiac disorders | Cardiac hypertrophy | 4 | 11.07  (4.15,29.56) | 11.07  (4.15,29.55) | 36.50 | 3.46  (0.58) | 11.03  (4.13) |
| Gastrointestinal disorders | Tongue oedema | 14 | 10.95  (6.48,18.52) | 10.95  (6.48,18.50) | 126.07 | 3.45  (1.97) | 10.91  (6.45) |
| Congenital, familial and genetic disorders | Congenital cardiovascular anomaly | 3 | 10.70  (3.44,33.24) | 10.69  (3.44,33.23) | 26.27 | 3.41  (0.20) | 10.66  (3.43) |
| Cardiac disorders | Cardiac failure acute | 23 | 10.62  (7.05,16.00) | 10.61  (7.05,15.97) | 199.47 | 3.40  (2.33) | 10.57  (7.02) |
| Investigations | Lymphocyte stimulation test positive | 3 | 10.42  (3.35,32.38) | 10.42  (3.35,32.37) | 25.46 | 3.38  (0.19) | 10.39  (3.34) |
| Renal and urinary disorders | Proteinuria | 62 | 10.26  (7.99,13.17) | 10.23  (7.98,13.13) | 514.84 | 3.35  (2.79) | 10.20  (7.95) |
| Skin and subcutaneous tissue disorders | Angioedema | 155 | 10.12  (8.64,11.85) | 10.05  (8.59,11.76) | 1259.41 | 3.32  (3.01) | 10.02  (8.55) |
| Renal and urinary disorders | Azotaemia | 13 | 9.94  (5.77,17.15) | 9.94  (5.77,17.13) | 104.15 | 3.31  (1.83) | 9.91  (5.75) |
| Congenital, familial and genetic disorders | Congenital musculoskeletal disorder of limbs | 3 | 9.89  (3.18,30.73) | 9.89  (3.18,30.72) | 23.89 | 3.30  (0.17) | 9.86  (3.17) |
| Cardiac disorders | Diastolic dysfunction | 10 | 9.86  (5.30,18.36) | 9.86  (5.30,18.34) | 79.35 | 3.30  (1.57) | 9.83  (5.28) |
| Investigations | Electrocardiogram T wave abnormal | 5 | 9.64  (4.01,23.19) | 9.64  (4.01,23.18) | 38.57 | 3.26  (0.80) | 9.61  (3.99) |
| Cardiac disorders | Angina pectoris | 90 | 9.34  (7.59,11.49) | 9.30  (7.57,11.44) | 665.18 | 3.21  (2.78) | 9.28  (7.54) |
| Investigations | Urine output decreased | 27 | 9.21  (6.31,13.44) | 9.20  (6.31,13.42) | 196.66 | 3.20  (2.28) | 9.17  (6.28) |
| Investigations | Electrocardiogram QRS complex prolonged | 15 | 9.18  (5.53,15.24) | 9.17  (5.53,15.23) | 108.91 | 3.19  (1.88) | 9.15  (5.51) |
| Gastrointestinal disorders | Lip exfoliation | 4 | 9.16  (3.43,24.46) | 9.16  (3.43,24.45) | 28.99 | 3.19  (0.50) | 9.14  (3.42) |
| Investigations | Blood creatine increased | 13 | 8.88  (5.15,15.31) | 8.87  (5.15,15.29) | 90.54 | 3.15  (1.73) | 8.85  (5.13) |
| Infections and infestations | Orchitis | 3 | 8.69  (2.80,27.00) | 8.69  (2.80,26.99) | 20.36 | 3.12  (0.13) | 8.67  (2.79) |
| Metabolism and nutrition disorders | Hypovolaemia | 17 | 8.68  (5.39,13.97) | 8.67  (5.39,13.95) | 115.01 | 3.11  (1.92) | 8.65  (5.37) |
| Nervous system disorders | Hemiplegia | 24 | 8.65  (5.79,12.91) | 8.64  (5.79,12.89) | 161.62 | 3.11  (2.14) | 8.61  (5.77) |
| Respiratory, thoracic and mediastinal disorders | Acute pulmonary oedema | 16 | 8.55  (5.23,13.97) | 8.55  (5.23,13.96) | 106.30 | 3.09  (1.86) | 8.52  (5.22) |
| Investigations | Glycosylated haemoglobin increased | 70 | 8.46  (6.69,10.70) | 8.44  (6.67,10.66) | 457.65 | 3.07  (2.59) | 8.41  (6.65) |
| Hepatobiliary disorders | Congestive hepatopathy | 4 | 8.39  (3.15,22.40) | 8.39  (3.15,22.39) | 25.97 | 3.07  (0.47) | 8.37  (3.14) |
| Gastrointestinal disorders | Enterocolitis haemorrhagic | 4 | 7.95  (2.98,21.21) | 7.95  (2.98,21.20) | 24.22 | 2.99  (0.44) | 7.93  (2.97) |
| Investigations | Blood pressure increased | 395 | 7.94  (7.18,8.77) | 7.80  (7.08,8.60) | 2342.27 | 2.96  (2.79) | 7.78  (7.05) |
| Surgical and medical procedures | Endotracheal intubation | 8 | 7.93  (3.96,15.87) | 7.93  (3.96,15.86) | 48.29 | 2.98  (1.20) | 7.91  (3.95) |
| Nervous system disorders | Ischaemic cerebral infarction | 3 | 7.90  (2.54,24.52) | 7.89  (2.54,24.52) | 18.01 | 2.98  (0.09) | 7.88  (2.54) |
| Eye disorders | Conjunctival haemorrhage | 10 | 7.82  (4.20,14.54) | 7.81  (4.20,14.53) | 59.25 | 2.96  (1.40) | 7.79  (4.19) |
| Cardiac disorders | Supraventricular extrasystoles | 9 | 7.81  (4.06,15.03) | 7.81  (4.06,15.02) | 53.28 | 2.96  (1.30) | 7.79  (4.05) |
| Surgical and medical procedures | Vascular graft | 8 | 7.81  (3.90,15.63) | 7.80  (3.90,15.62) | 47.33 | 2.96  (1.19) | 7.79  (3.89) |
| Vascular disorders | Infarction | 18 | 7.76  (4.88,12.33) | 7.75  (4.88,12.31) | 105.61 | 2.95  (1.85) | 7.74  (4.87) |
| Investigations | Blood sodium increased | 7 | 7.71  (3.67,16.19) | 7.71  (3.67,16.19) | 40.76 | 2.94  (1.04) | 7.69  (3.66) |
| Gastrointestinal disorders | Tongue haemorrhage | 3 | 7.71  (2.48,23.95) | 7.71  (2.48,23.94) | 17.47 | 2.94  (0.08) | 7.69  (2.48) |
| Respiratory, thoracic and mediastinal disorders | Pharyngeal oedema | 41 | 7.68  (5.65,10.44) | 7.67  (5.65,10.42) | 237.25 | 2.94  (2.28) | 7.65  (5.63) |
| General disorders and administration site conditions | Non-cardiac chest pain | 8 | 7.31  (3.65,14.63) | 7.31  (3.65,14.62) | 43.45 | 2.87  (1.14) | 7.29  (3.64) |
| Nervous system disorders | Tongue biting | 5 | 7.25  (3.02,17.45) | 7.25  (3.02,17.44) | 26.89 | 2.86  (0.65) | 7.24  (3.01) |
| Eye disorders | Diabetic retinopathy | 8 | 7.15  (3.57,14.32) | 7.15  (3.57,14.31) | 42.21 | 2.83  (1.12) | 7.13  (3.56) |
| Cardiac disorders | Cardiac failure | 187 | 7.11  (6.16,8.22) | 7.06  (6.12,8.14) | 970.87 | 2.82  (2.56) | 7.04  (6.10) |
| Cardiac disorders | Coronary artery stenosis | 12 | 6.95  (3.94,12.25) | 6.95  (3.94,12.24) | 60.95 | 2.79  (1.45) | 6.93  (3.93) |
| Cardiac disorders | Bundle branch block left | 10 | 6.95  (3.74,12.93) | 6.95  (3.73,12.92) | 50.77 | 2.79  (1.30) | 6.93  (3.73) |
| Injury, poisoning and procedural complications | Post procedural haematoma | 4 | 6.93  (2.60,18.48) | 6.93  (2.60,18.47) | 20.23 | 2.79  (0.37) | 6.91  (2.59) |
| Cardiac disorders | Myocardial ischaemia | 27 | 6.89  (4.72,10.06) | 6.89  (4.72,10.04) | 135.53 | 2.78  (1.96) | 6.87  (4.71) |
| Renal and urinary disorders | Oliguria | 14 | 6.78  (4.01,11.46) | 6.78  (4.01,11.45) | 68.81 | 2.76  (1.54) | 6.77  (4.00) |
| Investigations | Blood pressure systolic decreased | 10 | 6.71  (3.60,12.47) | 6.70  (3.60,12.47) | 48.41 | 2.74  (1.27) | 6.69  (3.60) |
| Cardiac disorders | Angina unstable | 15 | 6.59  (3.97,10.94) | 6.59  (3.97,10.93) | 70.93 | 2.72  (1.56) | 6.57  (3.96) |
| Cardiac disorders | Cardiomegaly | 28 | 6.58  (4.54,9.53) | 6.57  (4.53,9.51) | 131.90 | 2.71  (1.92) | 6.56  (4.52) |
| General disorders and administration site conditions | Effusion | 3 | 6.54  (2.11,20.31) | 6.54  (2.11,20.30) | 14.05 | 2.71  (0.01) | 6.53  (2.10) |
| Cardiac disorders | Tricuspid valve incompetence | 17 | 6.54  (4.06,10.53) | 6.53  (4.06,10.51) | 79.51 | 2.71  (1.64) | 6.52  (4.05) |
| Surgical and medical procedures | Catheter placement | 4 | 6.48  (2.43,17.29) | 6.48  (2.43,17.28) | 18.50 | 2.69  (0.33) | 6.47  (2.42) |
| Cardiac disorders | Left ventricular dysfunction | 14 | 6.44  (3.81,10.88) | 6.44  (3.81,10.87) | 64.14 | 2.68  (1.49) | 6.42  (3.80) |
| Respiratory, thoracic and mediastinal disorders | Dyspnoea at rest | 6 | 6.43  (2.89,14.33) | 6.43  (2.89,14.33) | 27.46 | 2.68  (0.76) | 6.42  (2.88) |
| Cardiac disorders | Mitral valve incompetence | 24 | 6.32  (4.23,9.43) | 6.31  (4.23,9.42) | 107.04 | 2.66  (1.80) | 6.30  (4.22) |
| Cardiac disorders | Dilated cardiomyopathy | 11 | 6.28  (3.47,11.34) | 6.27  (3.47,11.34) | 48.67 | 2.65  (1.29) | 6.26  (3.47) |
| Gastrointestinal disorders | Odynophagia | 12 | 6.26  (3.55,11.03) | 6.26  (3.55,11.02) | 52.89 | 2.64  (1.35) | 6.25  (3.54) |
| General disorders and administration site conditions | Face oedema | 35 | 6.20  (4.45,8.64) | 6.19  (4.45,8.63) | 152.09 | 2.63  (1.95) | 6.18  (4.44) |
| Respiratory, thoracic and mediastinal disorders | Rales | 12 | 6.12  (3.47,10.78) | 6.12  (3.47,10.78) | 51.26 | 2.61  (1.33) | 6.11  (3.46) |
| Respiratory, thoracic and mediastinal disorders | Suffocation feeling | 5 | 6.06  (2.52,14.57) | 6.06  (2.52,14.56) | 21.06 | 2.60  (0.54) | 6.04  (2.51) |
| Eye disorders | Eyelid oedema | 24 | 6.05  (4.05,9.03) | 6.04  (4.05,9.02) | 100.85 | 2.59  (1.75) | 6.03  (4.04) |
| Nervous system disorders | Sensory loss | 16 | 6.04  (3.70,9.87) | 6.04  (3.70,9.86) | 67.10 | 2.59  (1.52) | 6.03  (3.69) |
| Vascular disorders | Aortic stenosis | 7 | 6.03  (2.87,12.65) | 6.02  (2.87,12.64) | 29.27 | 2.59  (0.86) | 6.01  (2.86) |
| Metabolism and nutrition disorders | Hyperphosphataemia | 4 | 5.95  (2.23,15.88) | 5.95  (2.23,15.87) | 16.44 | 2.57  (0.29) | 5.94  (2.23) |
| Metabolism and nutrition disorders | Hyperuricaemia | 8 | 5.93  (2.96,11.87) | 5.93  (2.96,11.86) | 32.72 | 2.57  (0.97) | 5.92  (2.96) |
| Investigations | Blood pressure diastolic increased | 9 | 5.92  (3.08,11.39) | 5.92  (3.08,11.38) | 36.72 | 2.56  (1.07) | 5.91  (3.07) |
| Renal and urinary disorders | Renal impairment | 160 | 5.90  (5.05,6.90) | 5.87  (5.03,6.85) | 645.36 | 2.55  (2.28) | 5.86  (5.01) |
| Vascular disorders | Blood pressure fluctuation | 47 | 5.89  (4.43,7.85) | 5.88  (4.42,7.83) | 190.20 | 2.55  (2.00) | 5.87  (4.41) |
| General disorders and administration site conditions | Concomitant disease aggravated | 12 | 5.87  (3.33,10.34) | 5.86  (3.33,10.33) | 48.33 | 2.55  (1.29) | 5.85  (3.32) |
| Cardiac disorders | Atrioventricular block second degree | 6 | 5.87  (2.63,13.07) | 5.87  (2.63,13.07) | 24.17 | 2.55  (0.70) | 5.86  (2.63) |
| Renal and urinary disorders | Renal mass | 4 | 5.86  (2.20,15.64) | 5.86  (2.20,15.63) | 16.10 | 2.55  (0.28) | 5.85  (2.19) |
| Nervous system disorders | Carotid artery stenosis | 7 | 5.85  (2.79,12.29) | 5.85  (2.79,12.28) | 28.09 | 2.55  (0.84) | 5.84  (2.78) |
| General disorders and administration site conditions | Generalised oedema | 23 | 5.75  (3.82,8.66) | 5.74  (3.82,8.64) | 89.94 | 2.52  (1.67) | 5.73  (3.81) |
| Blood and lymphatic system disorders | Nephrogenic anaemia | 9 | 5.64  (2.93,10.85) | 5.64  (2.93,10.85) | 34.30 | 2.49  (1.03) | 5.63  (2.93) |
| Cardiac disorders | Ventricular hypertrophy | 5 | 5.62  (2.34,13.52) | 5.62  (2.34,13.51) | 18.95 | 2.49  (0.49) | 5.61  (2.33) |
| General disorders and administration site conditions | Oedema peripheral | 228 | 5.61  (4.93,6.40) | 5.56  (4.89,6.33) | 853.18 | 2.47  (2.25) | 5.55  (4.87) |
| Investigations | Protein urine present | 9 | 5.60  (2.91,10.77) | 5.60  (2.91,10.76) | 33.92 | 2.48  (1.02) | 5.59  (2.91) |
| Cardiac disorders | Ischaemic cardiomyopathy | 6 | 5.57  (2.50,12.41) | 5.57  (2.50,12.40) | 22.44 | 2.47  (0.66) | 5.56  (2.49) |
| Investigations | Protein total decreased | 7 | 5.54  (2.64,11.63) | 5.54  (2.64,11.62) | 25.98 | 2.47  (0.80) | 5.53  (2.63) |
| Surgical and medical procedures | Cardiac pacemaker insertion | 11 | 5.53  (3.06,9.99) | 5.53  (3.06,9.98) | 40.70 | 2.46  (1.17) | 5.52  (3.05) |
| Cardiac disorders | Ventricular hypokinesia | 6 | 5.53  (2.48,12.31) | 5.52  (2.48,12.30) | 22.19 | 2.46  (0.65) | 5.52  (2.48) |
| Respiratory, thoracic and mediastinal disorders | Laryngospasm | 5 | 5.52  (2.29,13.27) | 5.52  (2.29,13.27) | 18.46 | 2.46  (0.47) | 5.51  (2.29) |
| Cardiac disorders | Ventricular extrasystoles | 19 | 5.41  (3.45,8.49) | 5.41  (3.45,8.48) | 68.15 | 2.43  (1.50) | 5.40  (3.44) |
| Cardiac disorders | Dilatation ventricular | 4 | 5.40  (2.03,14.41) | 5.40  (2.03,14.40) | 14.32 | 2.43  (0.23) | 5.39  (2.02) |
| Cardiac disorders | Atrioventricular block first degree | 8 | 5.39  (2.70,10.79) | 5.39  (2.69,10.79) | 28.56 | 2.43  (0.89) | 5.38  (2.69) |
| Renal and urinary disorders | Nephrotic syndrome | 12 | 5.35  (3.04,9.42) | 5.35  (3.03,9.42) | 42.32 | 2.42  (1.20) | 5.34  (3.03) |
| Vascular disorders | Arterial occlusive disease | 14 | 5.33  (3.16,9.01) | 5.33  (3.15,9.00) | 49.14 | 2.41  (1.30) | 5.32  (3.15) |
| Cardiac disorders | Ventricular tachycardia | 29 | 5.29  (3.68,7.62) | 5.29  (3.67,7.61) | 100.61 | 2.40  (1.68) | 5.28  (3.67) |
| Neoplasms benign, malignant and unspecified (incl cysts and polyps) | Thyroid neoplasm | 5 | 5.29  (2.20,12.72) | 5.29  (2.20,12.72) | 17.36 | 2.40  (0.44) | 5.28  (2.20) |
| Renal and urinary disorders | Renal cyst | 14 | 5.26  (3.11,8.88) | 5.25  (3.11,8.87) | 48.13 | 2.39  (1.29) | 5.25  (3.10) |
| Cardiac disorders | Left ventricular failure | 6 | 5.24  (2.35,11.67) | 5.24  (2.35,11.66) | 20.52 | 2.39  (0.61) | 5.23  (2.35) |
| Cardiac disorders | Arrhythmia | 82 | 5.18  (4.17,6.44) | 5.17  (4.16,6.41) | 275.32 | 2.37  (1.98) | 5.16  (4.15) |
| Neoplasms benign, malignant and unspecified (incl cysts and polyps) | Lung cancer metastatic | 5 | 5.11  (2.13,12.30) | 5.11  (2.13,12.29) | 16.51 | 2.35  (0.42) | 5.11  (2.12) |
| Cardiac disorders | Coronary artery disease | 53 | 5.04  (3.85,6.60) | 5.03  (3.84,6.58) | 170.82 | 2.33  (1.83) | 5.02  (3.83) |
| Gastrointestinal disorders | Swollen tongue | 52 | 5.04  (3.83,6.61) | 5.03  (3.83,6.59) | 167.45 | 2.33  (1.82) | 5.02  (3.82) |
| General disorders and administration site conditions | Crepitations | 5 | 5.00  (2.08,12.02) | 5.00  (2.08,12.01) | 15.95 | 2.32  (0.40) | 4.99  (2.07) |
| Cardiac disorders | Aortic valve incompetence | 7 | 4.91  (2.34,10.32) | 4.91  (2.34,10.31) | 21.78 | 2.29  (0.70) | 4.91  (2.34) |
| Neoplasms benign, malignant and unspecified (incl cysts and polyps) | Rectal cancer | 5 | 4.86  (2.02,11.68) | 4.85  (2.02,11.67) | 15.28 | 2.28  (0.38) | 4.85  (2.02) |
| Gastrointestinal disorders | Lip swelling | 53 | 4.85  (3.70,6.35) | 4.84  (3.70,6.34) | 161.30 | 2.27  (1.78) | 4.83  (3.69) |
| Nervous system disorders | Diabetic neuropathy | 8 | 4.83  (2.41,9.66) | 4.83  (2.41,9.66) | 24.24 | 2.27  (0.80) | 4.82  (2.41) |
| Metabolism and nutrition disorders | Diabetes mellitus | 121 | 4.76  (3.98,5.70) | 4.74  (3.97,5.66) | 357.03 | 2.24  (1.94) | 4.73  (3.96) |
| Metabolism and nutrition disorders | Hypertriglyceridaemia | 9 | 4.68  (2.43,9.01) | 4.68  (2.43,9.00) | 26.02 | 2.23  (0.86) | 4.68  (2.43) |
| Renal and urinary disorders | Renal failure | 209 | 4.68  (4.08,5.36) | 4.64  (4.06,5.31) | 597.58 | 2.21  (1.99) | 4.64  (4.05) |
| Investigations | Renal function test abnormal | 7 | 4.66  (2.22,9.79) | 4.66  (2.22,9.78) | 20.11 | 2.22  (0.65) | 4.66  (2.22) |
| Cardiac disorders | Acute coronary syndrome | 13 | 4.60  (2.67,7.93) | 4.60  (2.67,7.92) | 36.57 | 2.20  (1.10) | 4.59  (2.67) |
| General disorders and administration site conditions | Oedema | 82 | 4.60  (3.70,5.72) | 4.59  (3.70,5.69) | 229.86 | 2.20  (1.82) | 4.58  (3.69) |
| Nervous system disorders | Hemiparesis | 26 | 4.58  (3.12,6.73) | 4.58  (3.12,6.72) | 72.56 | 2.19  (1.46) | 4.57  (3.11) |
| Metabolism and nutrition disorders | Hyponatraemia | 83 | 4.47  (3.60,5.55) | 4.46  (3.60,5.53) | 222.41 | 2.15  (1.78) | 4.45  (3.59) |
| Renal and urinary disorders | Nephropathy | 14 | 4.46  (2.64,7.53) | 4.45  (2.64,7.52) | 37.46 | 2.15  (1.11) | 4.45  (2.63) |
| Investigations | Blood pressure decreased | 96 | 4.39  (3.59,5.36) | 4.37  (3.58,5.34) | 249.50 | 2.13  (1.78) | 4.37  (3.57) |
| Investigations | Gamma-glutamyltransferase increased | 32 | 4.29  (3.03,6.07) | 4.29  (3.03,6.06) | 80.54 | 2.10  (1.46) | 4.28  (3.03) |
| Vascular disorders | Hypertension | 295 | 4.28  (3.82,4.81) | 4.24  (3.78,4.75) | 731.08 | 2.08  (1.90) | 4.23  (3.77) |
| Cardiac disorders | Atrioventricular block | 11 | 4.28  (2.37,7.73) | 4.27  (2.37,7.72) | 27.56 | 2.09  (0.91) | 4.27  (2.36) |
| General disorders and administration site conditions | Sudden death | 15 | 4.17  (2.51,6.92) | 4.16  (2.51,6.91) | 36.03 | 2.06  (1.07) | 4.16  (2.51) |
| Cardiac disorders | Atrial flutter | 11 | 4.12  (2.28,7.44) | 4.12  (2.28,7.44) | 25.92 | 2.04  (0.87) | 4.11  (2.28) |
| Gastrointestinal disorders | Large intestine polyp | 12 | 4.09  (2.32,7.21) | 4.09  (2.32,7.20) | 27.96 | 2.03  (0.92) | 4.08  (2.32) |
| Surgical and medical procedures | Dialysis | 19 | 4.01  (2.56,6.29) | 4.01  (2.56,6.29) | 42.86 | 2.00  (1.15) | 4.00  (2.55) |
| Nervous system disorders | Cerebral infarction | 32 | 3.92  (2.77,5.54) | 3.91  (2.77,5.53) | 69.34 | 1.97  (1.34) | 3.91  (2.76) |
| Cardiac disorders | Cardiogenic shock | 18 | 3.91  (2.47,6.22) | 3.91  (2.46,6.21) | 38.97 | 1.97  (1.10) | 3.91  (2.46) |
| Cardiac disorders | Ventricular fibrillation | 14 | 3.81  (2.25,6.43) | 3.80  (2.25,6.42) | 28.89 | 1.93  (0.93) | 3.80  (2.25) |
| Investigations | Electrocardiogram abnormal | 10 | 3.77  (2.03,7.01) | 3.77  (2.03,7.00) | 20.30 | 1.91  (0.72) | 3.76  (2.02) |
| Investigations | Haematocrit decreased | 25 | 3.71  (2.50,5.49) | 3.70  (2.50,5.48) | 49.27 | 1.89  (1.18) | 3.70  (2.50) |
| Renal and urinary disorders | Renal disorder | 57 | 3.70  (2.86,4.80) | 3.70  (2.85,4.79) | 112.03 | 1.88  (1.44) | 3.69  (2.85) |
| Respiratory, thoracic and mediastinal disorders | Sleep apnoea syndrome | 23 | 3.64  (2.42,5.48) | 3.64  (2.42,5.47) | 43.94 | 1.86  (1.12) | 3.63  (2.41) |
| Nervous system disorders | Motor dysfunction | 12 | 3.63  (2.06,6.40) | 3.63  (2.06,6.39) | 22.84 | 1.86  (0.79) | 3.63  (2.06) |
| Respiratory, thoracic and mediastinal disorders | Pulmonary congestion | 16 | 3.62  (2.22,5.91) | 3.62  (2.22,5.91) | 30.29 | 1.85  (0.95) | 3.62  (2.21) |
| Renal and urinary disorders | Chronic kidney disease | 102 | 3.61  (2.97,4.38) | 3.59  (2.96,4.36) | 190.81 | 1.84  (1.52) | 3.59  (2.95) |
| General disorders and administration site conditions | Swelling face | 77 | 3.60  (2.88,4.50) | 3.59  (2.87,4.49) | 143.75 | 1.84  (1.47) | 3.59  (2.87) |
| Investigations | Blood sodium decreased | 22 | 3.52  (2.32,5.35) | 3.52  (2.32,5.34) | 39.63 | 1.81  (1.06) | 3.52  (2.31) |
| Cardiac disorders | Bradycardia | 61 | 3.44  (2.68,4.43) | 3.44  (2.67,4.41) | 105.30 | 1.78  (1.36) | 3.43  (2.67) |
| Investigations | Heart rate decreased | 40 | 3.41  (2.50,4.65) | 3.40  (2.50,4.64) | 67.78 | 1.76  (1.23) | 3.40  (2.49) |
| Cardiac disorders | Acute myocardial infarction | 34 | 3.39  (2.42,4.75) | 3.39  (2.42,4.74) | 57.23 | 1.76  (1.18) | 3.39  (2.42) |
| Vascular disorders | Circulatory collapse | 19 | 3.34  (2.13,5.23) | 3.33  (2.13,5.23) | 31.02 | 1.74  (0.93) | 3.33  (2.12) |
| Infections and infestations | Gastroenteritis | 16 | 3.32  (2.04,5.43) | 3.32  (2.04,5.42) | 25.96 | 1.73  (0.85) | 3.32  (2.03) |
| Respiratory, thoracic and mediastinal disorders | Pulmonary oedema | 49 | 3.29  (2.48,4.35) | 3.28  (2.48,4.34) | 77.62 | 1.71  (1.24) | 3.28  (2.48) |
| Cardiac disorders | Atrial fibrillation | 105 | 3.27  (2.70,3.96) | 3.25  (2.69,3.94) | 164.03 | 1.70  (1.39) | 3.25  (2.68) |
| Nervous system disorders | Syncope | 109 | 3.26  (2.70,3.94) | 3.25  (2.70,3.92) | 169.98 | 1.70  (1.39) | 3.25  (2.69) |
| Vascular disorders | Hypotension | 214 | 3.26  (2.85,3.73) | 3.24  (2.83,3.70) | 331.80 | 1.69  (1.48) | 3.24  (2.83) |
| Nervous system disorders | Cerebrovascular accident | 184 | 3.26  (2.82,3.77) | 3.24  (2.80,3.74) | 285.00 | 1.69  (1.46) | 3.23  (2.80) |
| Nervous system disorders | Dysarthria | 40 | 3.21  (2.35,4.37) | 3.20  (2.35,4.36) | 60.51 | 1.68  (1.15) | 3.20  (2.35) |
| Metabolism and nutrition disorders | Gout | 19 | 3.20  (2.04,5.03) | 3.20  (2.04,5.02) | 28.75 | 1.68  (0.88) | 3.20  (2.04) |
| Renal and urinary disorders | Acute kidney injury | 197 | 3.06  (2.66,3.52) | 3.04  (2.65,3.50) | 270.80 | 1.60  (1.38) | 3.04  (2.64) |
| Nervous system disorders | Transient ischaemic attack | 34 | 2.99  (2.14,4.19) | 2.99  (2.13,4.18) | 44.91 | 1.58  (1.01) | 2.98  (2.13) |
| Gastrointestinal disorders | Gastritis | 26 | 2.95  (2.01,4.33) | 2.95  (2.01,4.33) | 33.43 | 1.56  (0.90) | 2.95  (2.00) |

Note1:ranked by ROR

Note2:Signals are detected when all the following criteria are met:a ≥ 3, PRR ≥2 and Chi-Square ≥ 4, lower limit of 95% CI of ROR > 1, IC025 > 0, EBGM05 > 2.

TABLE 5 Signal strength of adverse events at the Preferred Term(PT) level ranked by EBGM

| **System Organ Class(SOC)** | **Preferred Term(PT)** | **Case reports** | **ROR (95% CI)** | **PRR (95% CI)** | **Chi Square** | **IC (IC025)** | **EBGM (EBGM05)** |
| --- | --- | --- | --- | --- | --- | --- | --- |
| Vascular disorders | Paradoxical pressor response | 3 | 775.96  (216.46,2781.64) | 775.84  (216.46,2780.82) | 1824.06 | 9.25  (0.37) | 609.81  (170.11) |
| Investigations | Urine albumin/creatinine ratio increased | 58 | 433.15  (328.50,571.14) | 431.93  (327.79,569.14) | 21649.0 | 8.55  (5.27) | 375.12  (284.49) |
| Congenital, familial and genetic disorders | Persistent cloaca | 4 | 344.89  (122.17,973.60) | 344.82  (122.17,973.23) | 1223.04 | 8.27  (0.93) | 307.65  (108.98) |
| Congenital, familial and genetic disorders | Urachal abnormality | 4 | 284.53  (101.79,795.34) | 284.48  (101.79,795.04) | 1027.20 | 8.02  (0.94) | 258.71  (92.55) |
| Investigations | Renin increased | 15 | 175.73  (104.30,296.07) | 175.60  (104.27,295.74) | 2452.73 | 7.37  (3.13) | 165.45  (98.20) |
| Congenital, familial and genetic disorders | Conjoined twins | 5 | 159.86  (64.93,393.55) | 159.82  (64.93,393.37) | 747.15 | 7.24  (1.32) | 151.37  (61.49) |
| General disorders and administration site conditions | Concomitant disease progression | 89 | 139.26  (112.52,172.35) | 138.65  (112.13,171.45) | 11598.0 | 7.05  (5.44) | 132.26  (106.86) |
| Vascular disorders | Blood pressure inadequately controlled | 185 | 93.62  (80.82,108.46) | 92.79  (80.20,107.35) | 16268.4 | 6.49  (5.71) | 89.89  (77.59) |
| Congenital, familial and genetic disorders | Congenital bladder anomaly | 4 | 86.88  (32.12,234.97) | 86.86  (32.12,234.88) | 329.44 | 6.40  (0.94) | 84.32  (31.18) |
| Renal and urinary disorders | Microalbuminuria | 20 | 79.43  (50.92,123.89) | 79.35  (50.89,123.72) | 1505.32 | 6.27  (3.42) | 77.23  (49.51) |
| Endocrine disorders | Primary hyperaldosteronism | 4 | 77.95  (28.87,210.51) | 77.94  (28.87,210.43) | 295.70 | 6.25  (0.94) | 75.89  (28.10) |
| Investigations | Angiocardiogram | 4 | 77.42  (28.67,209.06) | 77.41  (28.67,208.98) | 293.69 | 6.24  (0.94) | 75.38  (27.92) |
| Renal and urinary disorders | Nephroangiosclerosis | 4 | 74.88  (27.74,202.10) | 74.86  (27.74,202.02) | 284.03 | 6.19  (0.93) | 72.97  (27.03) |
| Investigations | Blood aldosterone increased | 4 | 70.69  (26.21,190.66) | 70.68  (26.21,190.59) | 268.11 | 6.11  (0.93) | 68.99  (25.58) |
| Congenital, familial and genetic disorders | Truncus arteriosus persistent | 4 | 66.56  (24.69,179.39) | 66.54  (24.69,179.32) | 252.33 | 6.02  (0.93) | 65.05  (24.13) |
| Investigations | Creatinine urine increased | 6 | 63.24  (28.16,142.02) | 63.22  (28.15,141.95) | 359.41 | 5.95  (1.57) | 61.86  (27.54) |
| Nervous system disorders | Neurologic neglect syndrome | 10 | 60.68  (32.43,113.55) | 60.66  (32.43,113.46) | 574.48 | 5.89  (2.35) | 59.41  (31.75) |
| Surgical and medical procedures | Percutaneous coronary intervention | 5 | 52.31  (21.59,126.69) | 52.29  (21.59,126.64) | 247.02 | 5.68  (1.26) | 51.37  (21.21) |
| Renal and urinary disorders | Renal artery stenosis | 23 | 49.32  (32.65,74.50) | 49.27  (32.63,74.39) | 1069.16 | 5.60  (3.43) | 48.45  (32.07) |
| Investigations | Albumin urine present | 6 | 48.64  (21.70,109.03) | 48.63  (21.70,108.97) | 275.19 | 5.58  (1.54) | 47.83  (21.34) |
| Congenital, familial and genetic disorders | Intestinal malrotation | 4 | 44.98  (16.75,120.80) | 44.98  (16.75,120.75) | 169.32 | 5.47  (0.89) | 44.29  (16.49) |
| Nervous system disorders | Carotid artery thrombosis | 11 | 44.22  (24.37,80.23) | 44.20  (24.37,80.16) | 457.33 | 5.44  (2.42) | 43.54  (24.00) |
| Renal and urinary disorders | Diabetic nephropathy | 30 | 40.60  (28.31,58.23) | 40.54  (28.28,58.12) | 1140.81 | 5.32  (3.62) | 39.99  (27.88) |
| Surgical and medical procedures | Coronary angioplasty | 5 | 33.32  (13.80,80.47) | 33.31  (13.80,80.43) | 154.89 | 5.04  (1.19) | 32.94  (13.64) |
| Respiratory, thoracic and mediastinal disorders | Pulmonary vascular disorder | 5 | 30.53  (12.65,73.70) | 30.52  (12.65,73.67) | 141.26 | 4.92  (1.18) | 30.21  (12.51) |
| Respiratory, thoracic and mediastinal disorders | Dyspnoea paroxysmal nocturnal | 7 | 27.74  (13.18,58.41) | 27.73  (13.18,58.38) | 178.65 | 4.78  (1.65) | 27.48  (13.05) |
| Psychiatric disorders | Adjustment disorder | 10 | 24.92  (13.37,46.45) | 24.91  (13.37,46.42) | 227.52 | 4.63  (2.09) | 24.70  (13.25) |
| Investigations | Venous pressure jugular increased | 3 | 24.39  (7.83,75.99) | 24.38  (7.83,75.97) | 66.70 | 4.60  (0.38) | 24.18  (7.76) |
| Cardiac disorders | Left ventricular hypertrophy | 32 | 22.53  (15.91,31.92) | 22.50  (15.89,31.85) | 652.29 | 4.48  (3.26) | 22.33  (15.77) |
| Reproductive system and breast disorders | Organic erectile dysfunction | 3 | 20.82  (6.69,64.82) | 20.82  (6.69,64.80) | 56.18 | 4.37  (0.35) | 20.67  (6.64) |
| Respiratory, thoracic and mediastinal disorders | Orthopnoea | 22 | 20.54  (13.50,31.25) | 20.52  (13.49,31.20) | 405.60 | 4.35  (2.86) | 20.38  (13.40) |
| Investigations | Blood pressure diastolic decreased | 48 | 19.65  (14.79,26.11) | 19.60  (14.76,26.03) | 841.79 | 4.28  (3.41) | 19.48  (14.66) |
| Investigations | Blood urea increased | 111 | 19.56  (16.22,23.59) | 19.46  (16.16,23.44) | 1931.30 | 4.27  (3.78) | 19.34  (16.04) |
| Respiratory, thoracic and mediastinal disorders | Nocturnal dyspnoea | 8 | 19.21  (9.58,38.51) | 19.21  (9.58,38.49) | 137.13 | 4.25  (1.70) | 19.08  (9.52) |
| Vascular disorders | Aortic disorder | 5 | 18.36  (7.62,44.23) | 18.35  (7.62,44.21) | 81.51 | 4.19  (1.05) | 18.24  (7.57) |
| General disorders and administration site conditions | Therapy responder | 3 | 18.32  (5.89,57.00) | 18.31  (5.89,56.99) | 48.79 | 4.19  (0.33) | 18.20  (5.85) |
| Investigations | Brain natriuretic peptide increased | 18 | 17.99  (11.32,28.61) | 17.98  (11.31,28.57) | 286.83 | 4.16  (2.58) | 17.87  (11.24) |
| Injury, poisoning and procedural complications | Heat illness | 4 | 17.76  (6.64,47.46) | 17.75  (6.64,47.44) | 62.84 | 4.14  (0.73) | 17.65  (6.60) |
| Renal and urinary disorders | Albuminuria | 3 | 17.67  (5.68,54.99) | 17.67  (5.68,54.97) | 46.89 | 4.13  (0.32) | 17.57  (5.65) |
| Nervous system disorders | Vascular dementia | 5 | 17.54  (7.28,42.27) | 17.54  (7.28,42.25) | 77.50 | 4.12  (1.04) | 17.44  (7.24) |
| Vascular disorders | Malignant hypertension | 5 | 17.16  (7.12,41.35) | 17.16  (7.12,41.33) | 75.63 | 4.09  (1.03) | 17.06  (7.08) |
| Infections and infestations | Vestibular neuronitis | 3 | 16.94  (5.44,52.69) | 16.93  (5.44,52.68) | 44.71 | 4.07  (0.31) | 16.84  (5.41) |
| Cardiac disorders | Bradyarrhythmia | 9 | 16.73  (8.69,32.22) | 16.72  (8.69,32.20) | 132.27 | 4.06  (1.78) | 16.63  (8.64) |
| Vascular disorders | Hypertensive crisis | 61 | 16.62  (12.92,21.39) | 16.57  (12.89,21.31) | 887.69 | 4.04  (3.35) | 16.48  (12.81) |
| Renal and urinary disorders | Renal atrophy | 7 | 16.50  (7.85,34.70) | 16.50  (7.85,34.68) | 101.33 | 4.04  (1.46) | 16.41  (7.80) |
| Investigations | Creatinine renal clearance decreased | 21 | 16.38  (10.66,25.16) | 16.36  (10.66,25.12) | 301.19 | 4.02  (2.65) | 16.27  (10.60) |
| General disorders and administration site conditions | Sudden cardiac death | 19 | 16.22  (10.33,25.46) | 16.20  (10.32,25.43) | 269.49 | 4.01  (2.55) | 16.12  (10.26) |
| Investigations | Blood potassium increased | 86 | 16.14  (13.05,19.96) | 16.08  (13.01,19.86) | 1209.49 | 4.00  (3.46) | 15.99  (12.93) |
| Metabolism and nutrition disorders | Hyperkalaemia | 177 | 15.61  (13.45,18.10) | 15.48  (13.36,17.93) | 2385.53 | 3.94  (3.61) | 15.40  (13.28) |
| Investigations | Blood creatinine increased | 335 | 15.69  (14.08,17.49) | 15.45  (13.89,17.19) | 4508.81 | 3.94  (3.72) | 15.38  (13.80) |
| Eye disorders | Periorbital oedema | 26 | 15.31  (10.41,22.52) | 15.29  (10.41,22.48) | 345.53 | 3.93  (2.76) | 15.22  (10.35) |
| Neoplasms benign, malignant and unspecified (incl cysts and polyps) | Colorectal adenoma | 7 | 15.24  (7.25,32.04) | 15.24  (7.25,32.02) | 92.62 | 3.92  (1.43) | 15.16  (7.21) |
| Investigations | Glomerular filtration rate decreased | 58 | 15.14  (11.69,19.61) | 15.10  (11.67,19.54) | 759.90 | 3.91  (3.22) | 15.03  (11.61) |
| Cardiac disorders | Cardiac failure chronic | 21 | 15.04  (9.79,23.09) | 15.02  (9.79,23.06) | 273.42 | 3.90  (2.58) | 14.95  (9.73) |
| Cardiac disorders | Tachyarrhythmia | 12 | 14.36  (8.14,25.33) | 14.36  (8.14,25.31) | 148.36 | 3.84  (2.02) | 14.29  (8.10) |
| Injury, poisoning and procedural complications | Coronary artery restenosis | 3 | 14.27  (4.59,44.39) | 14.27  (4.59,44.37) | 36.84 | 3.83  (0.28) | 14.21  (4.57) |
| Psychiatric disorders | Personality disorder | 12 | 14.25  (8.08,25.13) | 14.24  (8.08,25.11) | 147.01 | 3.83  (2.01) | 14.18  (8.04) |
| Renal and urinary disorders | Glomerulosclerosis | 3 | 14.11  (4.54,43.87) | 14.11  (4.54,43.86) | 36.35 | 3.81  (0.27) | 14.04  (4.52) |
| Cardiac disorders | Left atrial dilatation | 6 | 13.89  (6.23,30.99) | 13.89  (6.23,30.97) | 71.41 | 3.79  (1.19) | 13.83  (6.20) |
| Investigations | Blood albumin increased | 4 | 13.42  (5.02,35.85) | 13.42  (5.03,35.83) | 45.76 | 3.74  (0.65) | 13.36  (5.00) |
| Endocrine disorders | Hyperparathyroidism secondary | 11 | 12.93  (7.15,23.38) | 12.92  (7.15,23.36) | 120.43 | 3.69  (1.86) | 12.87  (7.11) |
| Renal and urinary disorders | Nephrosclerosis | 5 | 12.83  (5.33,30.89) | 12.83  (5.33,30.87) | 54.28 | 3.67  (0.93) | 12.77  (5.31) |
| Nervous system disorders | Tongue paralysis | 3 | 12.82  (4.12,39.84) | 12.81  (4.12,39.83) | 32.53 | 3.67  (0.25) | 12.76  (4.10) |
| General disorders and administration site conditions | Sense of oppression | 5 | 12.65  (5.25,30.45) | 12.64  (5.25,30.43) | 53.38 | 3.65  (0.92) | 12.59  (5.23) |
| Congenital, familial and genetic disorders | Cardiac septal defect | 3 | 12.65  (4.07,39.31) | 12.64  (4.07,39.30) | 32.03 | 3.65  (0.24) | 12.59  (4.05) |
| General disorders and administration site conditions | Gravitational oedema | 3 | 12.55  (4.04,39.02) | 12.55  (4.04,39.01) | 31.75 | 3.64  (0.24) | 12.50  (4.02) |
| Surgical and medical procedures | Resuscitation | 7 | 12.44  (5.92,26.14) | 12.44  (5.92,26.13) | 73.31 | 3.63  (1.33) | 12.39  (5.90) |
| Investigations | Blood uric acid increased | 23 | 12.23  (8.12,18.43) | 12.22  (8.12,18.40) | 235.97 | 3.61  (2.46) | 12.17  (8.08) |
| Investigations | Blood urea decreased | 5 | 11.91  (4.95,28.66) | 11.90  (4.95,28.65) | 49.73 | 3.57  (0.90) | 11.86  (4.93) |
| Neoplasms benign, malignant and unspecified (incl cysts and polyps) | Transitional cell carcinoma | 5 | 11.72  (4.87,28.21) | 11.72  (4.87,28.20) | 48.81 | 3.55  (0.89) | 11.67  (4.85) |
| Cardiac disorders | Aortic valve sclerosis | 3 | 11.71  (3.77,36.39) | 11.71  (3.77,36.38) | 29.26 | 3.54  (0.22) | 11.66  (3.75) |
| Cardiac disorders | Extrasystoles | 27 | 11.51  (7.88,16.79) | 11.49  (7.88,16.77) | 257.61 | 3.52  (2.51) | 11.45  (7.84) |
| Gastrointestinal disorders | Oedema mouth | 13 | 11.46  (6.65,19.77) | 11.46  (6.65,19.75) | 123.58 | 3.51  (1.94) | 11.41  (6.62) |
| Gastrointestinal disorders | Lip oedema | 16 | 11.44  (7.00,18.69) | 11.43  (7.00,18.67) | 151.64 | 3.51  (2.12) | 11.39  (6.97) |
| Infections and infestations | Tracheobronchitis | 3 | 11.41  (3.67,35.46) | 11.41  (3.67,35.45) | 28.38 | 3.51  (0.22) | 11.37  (3.66) |
| Investigations | Electrocardiogram repolarisation abnormality | 3 | 11.38  (3.66,35.37) | 11.38  (3.66,35.36) | 28.29 | 3.50  (0.21) | 11.34  (3.65) |
| Investigations | Blood pressure systolic increased | 73 | 11.39  (9.05,14.34) | 11.35  (9.02,14.28) | 686.54 | 3.50  (2.97) | 11.31  (8.98) |
| Respiratory, thoracic and mediastinal disorders | Stridor | 11 | 11.32  (6.26,20.47) | 11.31  (6.26,20.45) | 103.01 | 3.49  (1.77) | 11.27  (6.23) |
| Surgical and medical procedures | Implantable defibrillator insertion | 5 | 11.26  (4.68,27.11) | 11.26  (4.68,27.10) | 46.57 | 3.49  (0.87) | 11.22  (4.66) |
| Musculoskeletal and connective tissue disorders | Gouty arthritis | 3 | 11.11  (3.58,34.54) | 11.11  (3.58,34.53) | 27.50 | 3.47  (0.21) | 11.07  (3.56) |
| Cardiac disorders | Cardiac hypertrophy | 4 | 11.07  (4.15,29.56) | 11.07  (4.15,29.55) | 36.50 | 3.46  (0.58) | 11.03  (4.13) |
| Gastrointestinal disorders | Tongue oedema | 14 | 10.95  (6.48,18.52) | 10.95  (6.48,18.50) | 126.07 | 3.45  (1.97) | 10.91  (6.45) |
| Congenital, familial and genetic disorders | Congenital cardiovascular anomaly | 3 | 10.70  (3.44,33.24) | 10.69  (3.44,33.23) | 26.27 | 3.41  (0.20) | 10.66  (3.43) |
| Cardiac disorders | Cardiac failure acute | 23 | 10.62  (7.05,16.00) | 10.61  (7.05,15.97) | 199.47 | 3.40  (2.33) | 10.57  (7.02) |
| Investigations | Lymphocyte stimulation test positive | 3 | 10.42  (3.35,32.38) | 10.42  (3.35,32.37) | 25.46 | 3.38  (0.19) | 10.39  (3.34) |
| Renal and urinary disorders | Proteinuria | 62 | 10.26  (7.99,13.17) | 10.23  (7.98,13.13) | 514.84 | 3.35  (2.79) | 10.20  (7.95) |
| Skin and subcutaneous tissue disorders | Angioedema | 155 | 10.12  (8.64,11.85) | 10.05  (8.59,11.76) | 1259.41 | 3.32  (3.01) | 10.02  (8.55) |
| Renal and urinary disorders | Azotaemia | 13 | 9.94  (5.77,17.15) | 9.94  (5.77,17.13) | 104.15 | 3.31  (1.83) | 9.91  (5.75) |
| Congenital, familial and genetic disorders | Congenital musculoskeletal disorder of limbs | 3 | 9.89  (3.18,30.73) | 9.89  (3.18,30.72) | 23.89 | 3.30  (0.17) | 9.86  (3.17) |
| Cardiac disorders | Diastolic dysfunction | 10 | 9.86  (5.30,18.36) | 9.86  (5.30,18.34) | 79.35 | 3.30  (1.57) | 9.83  (5.28) |
| Investigations | Electrocardiogram T wave abnormal | 5 | 9.64  (4.01,23.19) | 9.64  (4.01,23.18) | 38.57 | 3.26  (0.80) | 9.61  (3.99) |
| Cardiac disorders | Angina pectoris | 90 | 9.34  (7.59,11.49) | 9.30  (7.57,11.44) | 665.18 | 3.21  (2.78) | 9.28  (7.54) |
| Investigations | Urine output decreased | 27 | 9.21  (6.31,13.44) | 9.20  (6.31,13.42) | 196.66 | 3.20  (2.28) | 9.17  (6.28) |
| Investigations | Electrocardiogram QRS complex prolonged | 15 | 9.18  (5.53,15.24) | 9.17  (5.53,15.23) | 108.91 | 3.19  (1.88) | 9.15  (5.51) |
| Gastrointestinal disorders | Lip exfoliation | 4 | 9.16  (3.43,24.46) | 9.16  (3.43,24.45) | 28.99 | 3.19  (0.50) | 9.14  (3.42) |
| Investigations | Blood creatine increased | 13 | 8.88  (5.15,15.31) | 8.87  (5.15,15.29) | 90.54 | 3.15  (1.73) | 8.85  (5.13) |
| Infections and infestations | Orchitis | 3 | 8.69  (2.80,27.00) | 8.69  (2.80,26.99) | 20.36 | 3.12  (0.13) | 8.67  (2.79) |
| Metabolism and nutrition disorders | Hypovolaemia | 17 | 8.68  (5.39,13.97) | 8.67  (5.39,13.95) | 115.01 | 3.11  (1.92) | 8.65  (5.37) |
| Nervous system disorders | Hemiplegia | 24 | 8.65  (5.79,12.91) | 8.64  (5.79,12.89) | 161.62 | 3.11  (2.14) | 8.61  (5.77) |
| Respiratory, thoracic and mediastinal disorders | Acute pulmonary oedema | 16 | 8.55  (5.23,13.97) | 8.55  (5.23,13.96) | 106.30 | 3.09  (1.86) | 8.52  (5.22) |
| Investigations | Glycosylated haemoglobin increased | 70 | 8.46  (6.69,10.70) | 8.44  (6.67,10.66) | 457.65 | 3.07  (2.59) | 8.41  (6.65) |
| Hepatobiliary disorders | Congestive hepatopathy | 4 | 8.39  (3.15,22.40) | 8.39  (3.15,22.39) | 25.97 | 3.07  (0.47) | 8.37  (3.14) |
| Gastrointestinal disorders | Enterocolitis haemorrhagic | 4 | 7.95  (2.98,21.21) | 7.95  (2.98,21.20) | 24.22 | 2.99  (0.44) | 7.93  (2.97) |
| Surgical and medical procedures | Endotracheal intubation | 8 | 7.93  (3.96,15.87) | 7.93  (3.96,15.86) | 48.29 | 2.98  (1.20) | 7.91  (3.95) |
| Nervous system disorders | Ischaemic cerebral infarction | 3 | 7.90  (2.54,24.52) | 7.89  (2.54,24.52) | 18.01 | 2.98  (0.09) | 7.88  (2.54) |
| Eye disorders | Conjunctival haemorrhage | 10 | 7.82  (4.20,14.54) | 7.81  (4.20,14.53) | 59.25 | 2.96  (1.40) | 7.79  (4.19) |
| Cardiac disorders | Supraventricular extrasystoles | 9 | 7.81  (4.06,15.03) | 7.81  (4.06,15.02) | 53.28 | 2.96  (1.30) | 7.79  (4.05) |
| Surgical and medical procedures | Vascular graft | 8 | 7.81  (3.90,15.63) | 7.80  (3.90,15.62) | 47.33 | 2.96  (1.19) | 7.79  (3.89) |
| Investigations | Blood pressure increased | 395 | 7.94  (7.18,8.77) | 7.80  (7.08,8.60) | 2342.27 | 2.96  (2.79) | 7.78  (7.05) |
| Vascular disorders | Infarction | 18 | 7.76  (4.88,12.33) | 7.75  (4.88,12.31) | 105.61 | 2.95  (1.85) | 7.74  (4.87) |
| Investigations | Blood sodium increased | 7 | 7.71  (3.67,16.19) | 7.71  (3.67,16.19) | 40.76 | 2.94  (1.04) | 7.69  (3.66) |
| Gastrointestinal disorders | Tongue haemorrhage | 3 | 7.71  (2.48,23.95) | 7.71  (2.48,23.94) | 17.47 | 2.94  (0.08) | 7.69  (2.48) |
| Respiratory, thoracic and mediastinal disorders | Pharyngeal oedema | 41 | 7.68  (5.65,10.44) | 7.67  (5.65,10.42) | 237.25 | 2.94  (2.28) | 7.65  (5.63) |
| General disorders and administration site conditions | Non-cardiac chest pain | 8 | 7.31  (3.65,14.63) | 7.31  (3.65,14.62) | 43.45 | 2.87  (1.14) | 7.29  (3.64) |
| Nervous system disorders | Tongue biting | 5 | 7.25  (3.02,17.45) | 7.25  (3.02,17.44) | 26.89 | 2.86  (0.65) | 7.24  (3.01) |
| Eye disorders | Diabetic retinopathy | 8 | 7.15  (3.57,14.32) | 7.15  (3.57,14.31) | 42.21 | 2.83  (1.12) | 7.13  (3.56) |
| Cardiac disorders | Cardiac failure | 187 | 7.11  (6.16,8.22) | 7.06  (6.12,8.14) | 970.87 | 2.82  (2.56) | 7.04  (6.10) |
| Cardiac disorders | Coronary artery stenosis | 12 | 6.95  (3.94,12.25) | 6.95  (3.94,12.24) | 60.95 | 2.79  (1.45) | 6.93  (3.93) |
| Cardiac disorders | Bundle branch block left | 10 | 6.95  (3.74,12.93) | 6.95  (3.73,12.92) | 50.77 | 2.79  (1.30) | 6.93  (3.73) |
| Injury, poisoning and procedural complications | Post procedural haematoma | 4 | 6.93  (2.60,18.48) | 6.93  (2.60,18.47) | 20.23 | 2.79  (0.37) | 6.91  (2.59) |
| Cardiac disorders | Myocardial ischaemia | 27 | 6.89  (4.72,10.06) | 6.89  (4.72,10.04) | 135.53 | 2.78  (1.96) | 6.87  (4.71) |
| Renal and urinary disorders | Oliguria | 14 | 6.78  (4.01,11.46) | 6.78  (4.01,11.45) | 68.81 | 2.76  (1.54) | 6.77  (4.00) |
| Investigations | Blood pressure systolic decreased | 10 | 6.71  (3.60,12.47) | 6.70  (3.60,12.47) | 48.41 | 2.74  (1.27) | 6.69  (3.60) |
| Cardiac disorders | Angina unstable | 15 | 6.59  (3.97,10.94) | 6.59  (3.97,10.93) | 70.93 | 2.72  (1.56) | 6.57  (3.96) |
| Cardiac disorders | Cardiomegaly | 28 | 6.58  (4.54,9.53) | 6.57  (4.53,9.51) | 131.90 | 2.71  (1.92) | 6.56  (4.52) |
| General disorders and administration site conditions | Effusion | 3 | 6.54  (2.11,20.31) | 6.54  (2.11,20.30) | 14.05 | 2.71  (0.01) | 6.53  (2.10) |
| Cardiac disorders | Tricuspid valve incompetence | 17 | 6.54  (4.06,10.53) | 6.53  (4.06,10.51) | 79.51 | 2.71  (1.64) | 6.52  (4.05) |
| Surgical and medical procedures | Catheter placement | 4 | 6.48  (2.43,17.29) | 6.48  (2.43,17.28) | 18.50 | 2.69  (0.33) | 6.47  (2.42) |
| Cardiac disorders | Left ventricular dysfunction | 14 | 6.44  (3.81,10.88) | 6.44  (3.81,10.87) | 64.14 | 2.68  (1.49) | 6.42  (3.80) |
| Respiratory, thoracic and mediastinal disorders | Dyspnoea at rest | 6 | 6.43  (2.89,14.33) | 6.43  (2.89,14.33) | 27.46 | 2.68  (0.76) | 6.42  (2.88) |
| Cardiac disorders | Mitral valve incompetence | 24 | 6.32  (4.23,9.43) | 6.31  (4.23,9.42) | 107.04 | 2.66  (1.80) | 6.30  (4.22) |
| Cardiac disorders | Dilated cardiomyopathy | 11 | 6.28  (3.47,11.34) | 6.27  (3.47,11.34) | 48.67 | 2.65  (1.29) | 6.26  (3.47) |
| Gastrointestinal disorders | Odynophagia | 12 | 6.26  (3.55,11.03) | 6.26  (3.55,11.02) | 52.89 | 2.64  (1.35) | 6.25  (3.54) |
| General disorders and administration site conditions | Face oedema | 35 | 6.20  (4.45,8.64) | 6.19  (4.45,8.63) | 152.09 | 2.63  (1.95) | 6.18  (4.44) |
| Respiratory, thoracic and mediastinal disorders | Rales | 12 | 6.12  (3.47,10.78) | 6.12  (3.47,10.78) | 51.26 | 2.61  (1.33) | 6.11  (3.46) |
| Respiratory, thoracic and mediastinal disorders | Suffocation feeling | 5 | 6.06  (2.52,14.57) | 6.06  (2.52,14.56) | 21.06 | 2.60  (0.54) | 6.04  (2.51) |
| Eye disorders | Eyelid oedema | 24 | 6.05  (4.05,9.03) | 6.04  (4.05,9.02) | 100.85 | 2.59  (1.75) | 6.03  (4.04) |
| Nervous system disorders | Sensory loss | 16 | 6.04  (3.70,9.87) | 6.04  (3.70,9.86) | 67.10 | 2.59  (1.52) | 6.03  (3.69) |
| Vascular disorders | Aortic stenosis | 7 | 6.03  (2.87,12.65) | 6.02  (2.87,12.64) | 29.27 | 2.59  (0.86) | 6.01  (2.86) |
| Metabolism and nutrition disorders | Hyperphosphataemia | 4 | 5.95  (2.23,15.88) | 5.95  (2.23,15.87) | 16.44 | 2.57  (0.29) | 5.94  (2.23) |
| Metabolism and nutrition disorders | Hyperuricaemia | 8 | 5.93  (2.96,11.87) | 5.93  (2.96,11.86) | 32.72 | 2.57  (0.97) | 5.92  (2.96) |
| Investigations | Blood pressure diastolic increased | 9 | 5.92  (3.08,11.39) | 5.92  (3.08,11.38) | 36.72 | 2.56  (1.07) | 5.91  (3.07) |
| Vascular disorders | Blood pressure fluctuation | 47 | 5.89  (4.43,7.85) | 5.88  (4.42,7.83) | 190.20 | 2.55  (2.00) | 5.87  (4.41) |
| Renal and urinary disorders | Renal impairment | 160 | 5.90  (5.05,6.90) | 5.87  (5.03,6.85) | 645.36 | 2.55  (2.28) | 5.86  (5.01) |
| Cardiac disorders | Atrioventricular block second degree | 6 | 5.87  (2.63,13.07) | 5.87  (2.63,13.07) | 24.17 | 2.55  (0.70) | 5.86  (2.63) |
| General disorders and administration site conditions | Concomitant disease aggravated | 12 | 5.87  (3.33,10.34) | 5.86  (3.33,10.33) | 48.33 | 2.55  (1.29) | 5.85  (3.32) |
| Renal and urinary disorders | Renal mass | 4 | 5.86  (2.20,15.64) | 5.86  (2.20,15.63) | 16.10 | 2.55  (0.28) | 5.85  (2.19) |
| Nervous system disorders | Carotid artery stenosis | 7 | 5.85  (2.79,12.29) | 5.85  (2.79,12.28) | 28.09 | 2.55  (0.84) | 5.84  (2.78) |
| General disorders and administration site conditions | Generalised oedema | 23 | 5.75  (3.82,8.66) | 5.74  (3.82,8.64) | 89.94 | 2.52  (1.67) | 5.73  (3.81) |
| Blood and lymphatic system disorders | Nephrogenic anaemia | 9 | 5.64  (2.93,10.85) | 5.64  (2.93,10.85) | 34.30 | 2.49  (1.03) | 5.63  (2.93) |
| Cardiac disorders | Ventricular hypertrophy | 5 | 5.62  (2.34,13.52) | 5.62  (2.34,13.51) | 18.95 | 2.49  (0.49) | 5.61  (2.33) |
| Investigations | Protein urine present | 9 | 5.60  (2.91,10.77) | 5.60  (2.91,10.76) | 33.92 | 2.48  (1.02) | 5.59  (2.91) |
| Cardiac disorders | Ischaemic cardiomyopathy | 6 | 5.57  (2.50,12.41) | 5.57  (2.50,12.40) | 22.44 | 2.47  (0.66) | 5.56  (2.49) |
| General disorders and administration site conditions | Oedema peripheral | 228 | 5.61  (4.93,6.40) | 5.56  (4.89,6.33) | 853.18 | 2.47  (2.25) | 5.55  (4.87) |
| Investigations | Protein total decreased | 7 | 5.54  (2.64,11.63) | 5.54  (2.64,11.62) | 25.98 | 2.47  (0.80) | 5.53  (2.63) |
| Surgical and medical procedures | Cardiac pacemaker insertion | 11 | 5.53  (3.06,9.99) | 5.53  (3.06,9.98) | 40.70 | 2.46  (1.17) | 5.52  (3.05) |
| Cardiac disorders | Ventricular hypokinesia | 6 | 5.53  (2.48,12.31) | 5.52  (2.48,12.30) | 22.19 | 2.46  (0.65) | 5.52  (2.48) |
| Respiratory, thoracic and mediastinal disorders | Laryngospasm | 5 | 5.52  (2.29,13.27) | 5.52  (2.29,13.27) | 18.46 | 2.46  (0.47) | 5.51  (2.29) |
| Cardiac disorders | Ventricular extrasystoles | 19 | 5.41  (3.45,8.49) | 5.41  (3.45,8.48) | 68.15 | 2.43  (1.50) | 5.40  (3.44) |
| Cardiac disorders | Dilatation ventricular | 4 | 5.40  (2.03,14.41) | 5.40  (2.03,14.40) | 14.32 | 2.43  (0.23) | 5.39  (2.02) |
| Cardiac disorders | Atrioventricular block first degree | 8 | 5.39  (2.70,10.79) | 5.39  (2.69,10.79) | 28.56 | 2.43  (0.89) | 5.38  (2.69) |
| Renal and urinary disorders | Nephrotic syndrome | 12 | 5.35  (3.04,9.42) | 5.35  (3.03,9.42) | 42.32 | 2.42  (1.20) | 5.34  (3.03) |
| Vascular disorders | Arterial occlusive disease | 14 | 5.33  (3.16,9.01) | 5.33  (3.15,9.00) | 49.14 | 2.41  (1.30) | 5.32  (3.15) |
| Neoplasms benign, malignant and unspecified (incl cysts and polyps) | Thyroid neoplasm | 5 | 5.29  (2.20,12.72) | 5.29  (2.20,12.72) | 17.36 | 2.40  (0.44) | 5.28  (2.20) |
| Cardiac disorders | Ventricular tachycardia | 29 | 5.29  (3.68,7.62) | 5.29  (3.67,7.61) | 100.61 | 2.40  (1.68) | 5.28  (3.67) |
| Renal and urinary disorders | Renal cyst | 14 | 5.26  (3.11,8.88) | 5.25  (3.11,8.87) | 48.13 | 2.39  (1.29) | 5.25  (3.10) |
| Cardiac disorders | Left ventricular failure | 6 | 5.24  (2.35,11.67) | 5.24  (2.35,11.66) | 20.52 | 2.39  (0.61) | 5.23  (2.35) |
| Cardiac disorders | Arrhythmia | 82 | 5.18  (4.17,6.44) | 5.17  (4.16,6.41) | 275.32 | 2.37  (1.98) | 5.16  (4.15) |
| Neoplasms benign, malignant and unspecified (incl cysts and polyps) | Lung cancer metastatic | 5 | 5.11  (2.13,12.30) | 5.11  (2.13,12.29) | 16.51 | 2.35  (0.42) | 5.11  (2.12) |
| Cardiac disorders | Coronary artery disease | 53 | 5.04  (3.85,6.60) | 5.03  (3.84,6.58) | 170.82 | 2.33  (1.83) | 5.02  (3.83) |
| Gastrointestinal disorders | Swollen tongue | 52 | 5.04  (3.83,6.61) | 5.03  (3.83,6.59) | 167.45 | 2.33  (1.82) | 5.02  (3.82) |
| General disorders and administration site conditions | Crepitations | 5 | 5.00  (2.08,12.02) | 5.00  (2.08,12.01) | 15.95 | 2.32  (0.40) | 4.99  (2.07) |
| Cardiac disorders | Aortic valve incompetence | 7 | 4.91  (2.34,10.32) | 4.91  (2.34,10.31) | 21.78 | 2.29  (0.70) | 4.91  (2.34) |
| Neoplasms benign, malignant and unspecified (incl cysts and polyps) | Rectal cancer | 5 | 4.86  (2.02,11.68) | 4.85  (2.02,11.67) | 15.28 | 2.28  (0.38) | 4.85  (2.02) |
| Gastrointestinal disorders | Lip swelling | 53 | 4.85  (3.70,6.35) | 4.84  (3.70,6.34) | 161.30 | 2.27  (1.78) | 4.83  (3.69) |
| Nervous system disorders | Diabetic neuropathy | 8 | 4.83  (2.41,9.66) | 4.83  (2.41,9.66) | 24.24 | 2.27  (0.80) | 4.82  (2.41) |
| Metabolism and nutrition disorders | Diabetes mellitus | 121 | 4.76  (3.98,5.70) | 4.74  (3.97,5.66) | 357.03 | 2.24  (1.94) | 4.73  (3.96) |
| Metabolism and nutrition disorders | Hypertriglyceridaemia | 9 | 4.68  (2.43,9.01) | 4.68  (2.43,9.00) | 26.02 | 2.23  (0.86) | 4.68  (2.43) |
| Investigations | Renal function test abnormal | 7 | 4.66  (2.22,9.79) | 4.66  (2.22,9.78) | 20.11 | 2.22  (0.65) | 4.66  (2.22) |
| Renal and urinary disorders | Renal failure | 209 | 4.68  (4.08,5.36) | 4.64  (4.06,5.31) | 597.58 | 2.21  (1.99) | 4.64  (4.05) |
| Cardiac disorders | Acute coronary syndrome | 13 | 4.60  (2.67,7.93) | 4.60  (2.67,7.92) | 36.57 | 2.20  (1.10) | 4.59  (2.67) |
| General disorders and administration site conditions | Oedema | 82 | 4.60  (3.70,5.72) | 4.59  (3.70,5.69) | 229.86 | 2.20  (1.82) | 4.58  (3.69) |
| Nervous system disorders | Hemiparesis | 26 | 4.58  (3.12,6.73) | 4.58  (3.12,6.72) | 72.56 | 2.19  (1.46) | 4.57  (3.11) |
| Metabolism and nutrition disorders | Hyponatraemia | 83 | 4.47  (3.60,5.55) | 4.46  (3.60,5.53) | 222.41 | 2.15  (1.78) | 4.45  (3.59) |
| Renal and urinary disorders | Nephropathy | 14 | 4.46  (2.64,7.53) | 4.45  (2.64,7.52) | 37.46 | 2.15  (1.11) | 4.45  (2.63) |
| Investigations | Blood pressure decreased | 96 | 4.39  (3.59,5.36) | 4.37  (3.58,5.34) | 249.50 | 2.13  (1.78) | 4.37  (3.57) |
| Investigations | Gamma-glutamyltransferase increased | 32 | 4.29  (3.03,6.07) | 4.29  (3.03,6.06) | 80.54 | 2.10  (1.46) | 4.28  (3.03) |
| Cardiac disorders | Atrioventricular block | 11 | 4.28  (2.37,7.73) | 4.27  (2.37,7.72) | 27.56 | 2.09  (0.91) | 4.27  (2.36) |
| Vascular disorders | Hypertension | 295 | 4.28  (3.82,4.81) | 4.24  (3.78,4.75) | 731.08 | 2.08  (1.90) | 4.23  (3.77) |
| General disorders and administration site conditions | Sudden death | 15 | 4.17  (2.51,6.92) | 4.16  (2.51,6.91) | 36.03 | 2.06  (1.07) | 4.16  (2.51) |
| Cardiac disorders | Atrial flutter | 11 | 4.12  (2.28,7.44) | 4.12  (2.28,7.44) | 25.92 | 2.04  (0.87) | 4.11  (2.28) |
| Gastrointestinal disorders | Large intestine polyp | 12 | 4.09  (2.32,7.21) | 4.09  (2.32,7.20) | 27.96 | 2.03  (0.92) | 4.08  (2.32) |
| Surgical and medical procedures | Dialysis | 19 | 4.01  (2.56,6.29) | 4.01  (2.56,6.29) | 42.86 | 2.00  (1.15) | 4.00  (2.55) |
| Nervous system disorders | Cerebral infarction | 32 | 3.92  (2.77,5.54) | 3.91  (2.77,5.53) | 69.34 | 1.97  (1.34) | 3.91  (2.76) |
| Cardiac disorders | Cardiogenic shock | 18 | 3.91  (2.47,6.22) | 3.91  (2.46,6.21) | 38.97 | 1.97  (1.10) | 3.91  (2.46) |
| Cardiac disorders | Ventricular fibrillation | 14 | 3.81  (2.25,6.43) | 3.80  (2.25,6.42) | 28.89 | 1.93  (0.93) | 3.80  (2.25) |
| Investigations | Electrocardiogram abnormal | 10 | 3.77  (2.03,7.01) | 3.77  (2.03,7.00) | 20.30 | 1.91  (0.72) | 3.76  (2.02) |
| Investigations | Haematocrit decreased | 25 | 3.71  (2.50,5.49) | 3.70  (2.50,5.48) | 49.27 | 1.89  (1.18) | 3.70  (2.50) |
| Renal and urinary disorders | Renal disorder | 57 | 3.70  (2.86,4.80) | 3.70  (2.85,4.79) | 112.03 | 1.88  (1.44) | 3.69  (2.85) |
| Respiratory, thoracic and mediastinal disorders | Sleep apnoea syndrome | 23 | 3.64  (2.42,5.48) | 3.64  (2.42,5.47) | 43.94 | 1.86  (1.12) | 3.63  (2.41) |
| Nervous system disorders | Motor dysfunction | 12 | 3.63  (2.06,6.40) | 3.63  (2.06,6.39) | 22.84 | 1.86  (0.79) | 3.63  (2.06) |
| Respiratory, thoracic and mediastinal disorders | Pulmonary congestion | 16 | 3.62  (2.22,5.91) | 3.62  (2.22,5.91) | 30.29 | 1.85  (0.95) | 3.62  (2.21) |
| Renal and urinary disorders | Chronic kidney disease | 102 | 3.61  (2.97,4.38) | 3.59  (2.96,4.36) | 190.81 | 1.84  (1.52) | 3.59  (2.95) |
| General disorders and administration site conditions | Swelling face | 77 | 3.60  (2.88,4.50) | 3.59  (2.87,4.49) | 143.75 | 1.84  (1.47) | 3.59  (2.87) |
| Investigations | Blood sodium decreased | 22 | 3.52  (2.32,5.35) | 3.52  (2.32,5.34) | 39.63 | 1.81  (1.06) | 3.52  (2.31) |
| Cardiac disorders | Bradycardia | 61 | 3.44  (2.68,4.43) | 3.44  (2.67,4.41) | 105.30 | 1.78  (1.36) | 3.43  (2.67) |
| Investigations | Heart rate decreased | 40 | 3.41  (2.50,4.65) | 3.40  (2.50,4.64) | 67.78 | 1.76  (1.23) | 3.40  (2.49) |
| Cardiac disorders | Acute myocardial infarction | 34 | 3.39  (2.42,4.75) | 3.39  (2.42,4.74) | 57.23 | 1.76  (1.18) | 3.39  (2.42) |
| Vascular disorders | Circulatory collapse | 19 | 3.34  (2.13,5.23) | 3.33  (2.13,5.23) | 31.02 | 1.74  (0.93) | 3.33  (2.12) |
| Infections and infestations | Gastroenteritis | 16 | 3.32  (2.04,5.43) | 3.32  (2.04,5.42) | 25.96 | 1.73  (0.85) | 3.32  (2.03) |
| Respiratory, thoracic and mediastinal disorders | Pulmonary oedema | 49 | 3.29  (2.48,4.35) | 3.28  (2.48,4.34) | 77.62 | 1.71  (1.24) | 3.28  (2.48) |
| Cardiac disorders | Atrial fibrillation | 105 | 3.27  (2.70,3.96) | 3.25  (2.69,3.94) | 164.03 | 1.70  (1.39) | 3.25  (2.68) |
| Nervous system disorders | Syncope | 109 | 3.26  (2.70,3.94) | 3.25  (2.70,3.92) | 169.98 | 1.70  (1.39) | 3.25  (2.69) |
| Vascular disorders | Hypotension | 214 | 3.26  (2.85,3.73) | 3.24  (2.83,3.70) | 331.80 | 1.69  (1.48) | 3.24  (2.83) |
| Nervous system disorders | Cerebrovascular accident | 184 | 3.26  (2.82,3.77) | 3.24  (2.80,3.74) | 285.00 | 1.69  (1.46) | 3.23  (2.80) |
| Metabolism and nutrition disorders | Gout | 19 | 3.20  (2.04,5.03) | 3.20  (2.04,5.02) | 28.75 | 1.68  (0.88) | 3.20  (2.04) |
| Nervous system disorders | Dysarthria | 40 | 3.21  (2.35,4.37) | 3.20  (2.35,4.36) | 60.51 | 1.68  (1.15) | 3.20  (2.35) |
| Renal and urinary disorders | Acute kidney injury | 197 | 3.06  (2.66,3.52) | 3.04  (2.65,3.50) | 270.80 | 1.60  (1.38) | 3.04  (2.64) |
| Nervous system disorders | Transient ischaemic attack | 34 | 2.99  (2.14,4.19) | 2.99  (2.13,4.18) | 44.91 | 1.58  (1.01) | 2.98  (2.13) |
| Gastrointestinal disorders | Gastritis | 26 | 2.95  (2.01,4.33) | 2.95  (2.01,4.33) | 33.43 | 1.56  (0.90) | 2.95  (2.00) |

Note1:ranked by EBGM

Note2:Signals are detected when all the following criteria are met:a ≥ 3, PRR ≥2 and Chi-Square ≥ 4, lower limit of 95% CI of ROR > 1, IC025 > 0, EBGM05 > 2.

TABLE 6 Time-to-onset analysis using the Weibull distribution test.

|  | | **Weibull distribution** | | | |  |
| --- | --- | --- | --- | --- | --- | --- |
| **Cases** | **TTO (days)** | **Scale parameter** | | **Shape parameter** | |  |
| **n** | **median(IQR)** | **α** | **95% CI** | **β** | **95% CI** | **Failure type** |
| 2209 | 62.00(7.00,282.00) | 180.19 | 167.38 - 193.97 | 0.64 | 0.62 - 0.66 | Early failure |
